# Understanding Uncertainties in Model-Based Predictions of *Aedes aegypti* Population Dynamics

**DOI:** 10.1371/journal.pntd.0000830

**Published:** 2010-09-28

**Authors:** Chonggang Xu, Mathieu Legros, Fred Gould, Alun L. Lloyd

**Affiliations:** 1 Department of Entomology, North Carolina State University, Raleigh, North Carolina, United States of America; 2 Department of Mathematics and Biomathematics Graduate Program, North Carolina State University, Raleigh, North Carolina, United States of America; The University of Queensland, Australia

## Abstract

**Background:**

*Aedes aegypti* is one of the most important mosquito vectors of human disease. The development of spatial models for *Ae. aegypti* provides a promising start toward model-guided vector control and risk assessment, but this will only be possible if models make reliable predictions. The reliability of model predictions is affected by specific sources of uncertainty in the model.

**Methodology/Principal Findings:**

This study quantifies uncertainties in the predicted mosquito population dynamics at the community level (a cluster of 612 houses) and the individual-house level based on Skeeter Buster, a spatial model of *Ae. aegypti*, for the city of Iquitos, Peru. The study considers two types of uncertainty: 1) uncertainty in the estimates of 67 parameters that describe mosquito biology and life history, and 2) uncertainty due to environmental and demographic stochasticity. Our results show that for pupal density and for female adult density at the community level, respectively, the 95% prediction confidence interval ranges from 1000 to 3000 and from 700 to 5,000 individuals. The two parameters contributing most to the uncertainties in predicted population densities at both individual-house and community levels are the female adult survival rate and a coefficient determining weight loss due to energy used in metabolism at the larval stage (i.e. metabolic weight loss). Compared to parametric uncertainty, stochastic uncertainty is relatively low for population density predictions at the community level (less than 5% of the overall uncertainty) but is substantially higher for predictions at the individual-house level (larger than 40% of the overall uncertainty). Uncertainty in mosquito spatial dispersal has little effect on population density predictions at the community level but is important for the prediction of spatial clustering at the individual-house level.

**Conclusion/Significance:**

This is the first systematic uncertainty analysis of a detailed *Ae. aegypti* population dynamics model and provides an approach for identifying those parameters for which more accurate estimates would improve model predictions.

## Author Summary

Dengue is one of the most important insect-vectored human viral diseases. The principal vector is *Aedes aegypti*, a mosquito that lives in close association with humans. Currently, there is no effective vaccine available and the only means for limiting dengue outbreaks is vector control. To help design vector control strategies, spatial models of *Ae. aegypti* population dynamics have been developed. However, the usefulness of such models depends on the reliability of their predictions, which can be affected by different sources of uncertainty including uncertainty in the model parameter estimation, uncertainty in the model structure, measurement errors in the data fed into the model, individual variability, and stochasticity in the environment. This study quantifies uncertainties in the mosquito population dynamics predicted by Skeeter Buster, a spatial model of *Ae. aegypti*, for the city of Iquitos, Peru. The uncertainty quantification should enable us to better understand the reliability of model predictions, improve Skeeter Buster and other similar models by targeting those parameters with high uncertainty contributions for further empirical research, and thereby decrease uncertainty in model predictions.

## Introduction

*Aedes aegypti* is one of the most important mosquito vectors of human viral diseases. It causes approximately 50 million cases of dengue fever per year, 500,000 cases of dengue hemorrhagic fever (DHF) or dengue shock syndrome (DSS), and approximately 12,500 fatalities annually [1], [2]. Currently, there is no effective vaccine available and the only means for limiting dengue outbreaks is vector control. For a better understanding of mosquito population dynamics and more efficient vector and disease control, researchers have built mathematical models that incorporate fundamental biological and ecological mechanisms affecting mosquito population dynamics. A pioneering model was developed by Gilpin & McClelland [3] to predict how larval development is affected by food density, larval weight and temperature. Although Gilpin & McClelland's model was based on larvae in an artificial laboratory environment and did not simulate the whole life cycle of *Ae. aegypti*, their model was significant in providing the first biologically realistic approach for predicting larval population dynamics.

Based on Gilpin & McClelland's model, Focks et al. [4] developed a life history model (CIMSiM) to predict in-field population dynamics for *Ae. aegypti*. This model incorporated detailed biological processes (survival, physiological developments, food-regulated body weight growth, and fecundities) and environmental factors (temperature and humidity) for four different life stages: eggs, larvae, pupae and adults. It has been applied to a number of villages and city environments, including locations in Thailand and the US [5]. By coupling CIMSiM with an epidemiological simulation model (DENSiM), it is possible to make predictions about disease dynamics [6]. The model has also been scaled up to global levels to predict the potential effects of climatic change on mosquito population distributions and potential disease risks [7].

The CIMSiM model does not account for spatial heterogeneities in the mosquito population and its environment, and the dispersal of mosquitoes across this environment [8]. Recently, in view of the potential importance of spatial dispersal for mosquito population dynamics and vector control [9], [10], new spatial models have been developed [11], [12]. Based on their spatial model, Otero et al. [11] predicted that dispersal could be a significant factor impacting the seasonal population dynamics of *Ae. aegypti* in Buenos Aires, Argentina where the environment is marginal for this mosquito species. Magori et al. [12], using the stochastic and spatially explicit Skeeter Buster model, predicted that dispersal among houses would decrease spatial variations in mosquito densities caused by heterogeneity in the larval habitats among houses in tropical areas. Results from the Skeeter Buster model [12] also indicated that dispersal could impact the efficiency of some transgenic approaches for replacing native mosquito genotypes with engineered genotypes that do not transmit dengue [13], [14], [15].

Spatial models of *Ae. aegypti* could provide an important advance toward model-guided vector control and risk assessment. Attempts to compare the outcomes of different types of control strategies (e.g., physical removal of breeding sites, chemical control using adulticidal spraying of houses or larvicidal treatment of water-filled containers, and biological control of releasing transgenic mosquitoes for replacing native mosquito genotypes), used either in isolation or in combination, may require the use of models that include detailed descriptions of underlying biological processes. As a result, complex models are being increasingly used in disease and population modeling contexts. However, such models are analytically less tractable than their simple counterparts and can have many different sources of uncertainties, which may affect the reliability of predictions. There are four types of uncertainty in a model [16], [17]: 1) uncertainty in the model structure; 2) uncertainty in the parameter estimates; 3) uncertainties in data inputs for the model; and 4) stochastic uncertainty (i.e., the variability that results from environmental and demographic stochasticity). The first three types of uncertainty are generally reducible to some extent (*i.e.* uncertainty can be reduced given higher quality data and a better understanding of the system being simulated), while stochastic uncertainty is generally irreducible [18]. It is possible that the combination of these uncertainties will result in model predictions that are less reliable than acceptable to researchers and practitioners working to suppress dengue. This makes uncertainty analysis indispensible for complex models.

To evaluate the reliability of predictions made by Skeeter Buster model, we quantify uncertainties in the predicted *Ae. aegypti* population dynamics at the community level (a cluster of 612 houses) and the individual-house level. We focus on uncertainties in model predictions resulting from parametric uncertainty and stochasticity. In addition to quantifying overall parametric uncertainty, we also quantify proportions of uncertainty in model predictions contributed by specific model parameters using an advanced uncertainty analysis technique, the improved Fourier Amplitude Sensitivity Test (FAST) [19], [20], [21]. This should enable a better understanding of the factors contributing to uncertainty, and could enable targeting of parameters with high uncertainty contributions for more accurate empirical quantification. Although uncertainties in model structures and data inputs could also be important, it would be difficult to estimate them with currently available information.

## Materials and Methods

### Model description

In this section, we only provide an overview of Skeeter Buster. For a more detailed description of the model see Magori et al. [12]. Skeeter Buster simulates the biological development of four life stages of *Ae. aegypti*: eggs, larvae, pupae and adults. The model assumes that larval growth and survival are regulated by the amount of food available in water-filled containers in and around houses. The time from egg hatch to pupation and the period taken for each gonotrophic cycle (the egg production/laying cycles of female adults) are mainly determined by temperature-dependent development rates [22]. The pupation time also depends on larval weight, which is calculated using a weight gain model based on the work of Gilpin and McClelland [3]. Fecundity is assumed to be related to female adult weight [4], [23] (see Table S1). The survivorship of each life stage is dependent on temperature (see Figure S1), and survivorship of adults and eggs is also dependent on humidity (see Figure S2). The daily survival probability within the optimum range of environmental factors is termed the nominal survival rate. Egg hatching is dependent on water level change in the container. Skeeter Buster tracks the water temperature and water level for all containers based on container characteristics (e.g., size of opening), precipitation and air temperature.

### Study area description

In this study, we use environmental and spatial habitat data from the city of Iquitos, Peru (see Figure S3 for air temperature inputs) as a follow-up to Legros et al. [24] that uses data from this city to examine predictions of the basic model. Detailed descriptions of the study area have been provided in earlier studies [25], [26]. A mosquito survey using four-month long sampling circuits within the city, linked to a geographic information system, has been conducted since 1998. The survey recorded the proportion of water-filled containers holding pupae, the number of pupae, and the number of captured adults [25].

We simulate a district in the city with 612 houses as in Legros et al. [24]. Food inputs for different types of water containers are parameterized so that pupal densities simulated by Skeeter Buster (using default parameter values in Table S1, S2, S3, S4, S5, the assumed most likely values based on data and experiences) fit pupal data in the mosquito survey conducted in Iquitos [24] (see Figure S4 for the food input map). We initiate the model with 20 eggs for every container and run the model for 3 months to allow mosquito population dynamics to stabilize. The container water temperatures are simulated using a polynomial function obtained from a regression of water temperature on air temperature and sun exposure for 12 containers monitored for 76 days in Gainesville, FL, USA [4]. We rely on these data because similar information for Iquitos is lacking.

### Uncertainty analysis

The first step in our analysis involved assessment of both literature and expert knowledge to gauge the level of uncertainty related to values of each parameter. For the use of expert knowledge, we conducted workshops in 2008 and 2009 that included members of our own lab and two other mosquito ecology labs: Professor Thomas Scott's Lab (University of California, Davis) and Professor Laura Harrington's Lab (Cornell University). We selected individuals from these three labs because they have been working on *Ae. aegypti* for many years and because they are familiar with the modeling framework that we are using. Details of our elicitation process are given in Text S1. Please see Table 1 for definitions of uncertainty for those parameters that our analyses identify as being most important. A complete list of uncertainties for all parameters considered in our analyses is presented in Tables S1, S2, S3, S4, S5.

**Table 1:** Uncertainties in the estimates of parameters.

Parameter	Description	Lower Range	Upper Range	*Default Value*	*Confidence level for default value^1^*
*A-FS*	Nominal survival rate for female adults	0.75	0.99	0.89	Moderate
*A-MS*	Nominal survival rate for male adults	0.72	0.99	0.77	Moderate
A-*F*	Coefficient of fecundity for female adults^2^	35	55	46.5	Low
*E-PTH*	High temperature limit for predator activities on eggs (°C)^3^	25	35	30	Low
*E-SPTH*	Survival factor of predation at high temperatures for eggs^4^	0.65	0.9	0.7	Low
*Fc*	Coefficient of food dependence for larvae^5^	0.05	1	0.1	No
*Fd1*	Coefficient of metabolic weight loss for larvae^6^	0.005	0.032	0.016	Low
*L-D*	Larval development rate^7^	N/A			
*L-S*	Nominal survival rate for larvae	0.9	1	0.99	Low
*SD-FL*	Long-range dispersal probability for female adults	0	0.1	0.02	Low
*SD-FS*	Short-range dispersal probability for female adults	0.05	0.5	0.3	Low

Note 1: This level determines the probability density function defined between lower and upper range. Higher confidence level indicates higher probability around the default parameter value. See Text S1 for details; 2: unit: number of eggs per mg wet-weight of female adults. 3: This is the temperature above which predator activities increase. 4: The adjustment factor for survival due to predation if the temperature is higher than the specified high temperature limit for predator activities. 5: This coefficient specifies the effect of food amount on larval weight gain, with a lower value indicating a stronger effect of food on larval growth and higher level of density dependence (see Text S2 for a more detailed explanation). 6:This coefficient determines weight loss due to calories used in metabolism at larval stage. Its uncertainty range is defined such that the percent of weight loss due to metabolic activities is between 0.5 and 3.2 percent of body weight gain with no food constraint. See Text S2 for details. 7: The larval development rate determines the enzyme-controlled development of larvae, which is dependent on temperatures. The uncertainty range of this parameter is determined by fitting the model to data. See Text S1.3 and Text S3 for a more detailed explanation.

Here we only list the most important parameters, as identified by our uncertainty analyses. A complete list appears in Tables S1, S2, S3, S4, S5.

Many parametric uncertainty analysis techniques are now available [27], [28]. One of the most popular parametric uncertainty analysis techniques is FAST [29], [30], [31], which uses a periodic sampling approach and a Fourier transformation to quantify uncertainties in model predictions as measured by the variances and decomposes the total variance of a model output into partial variances contributed by individual model parameters. Ratios of partial variances to the total variance are used to measure the importance of parameters in their contributions to uncertainties in model predictions. The FAST analysis is a first-order global sensitivity analysis method for linear/nonlinear models that quantifies the separate contribution of each parameter to uncertainty, averaging over the values of all other parameters. These main effects do not consider the combined effects of two or more parameters. The traditional version of FAST assumes independence among parameters, but in this study, we used an improved version of FAST developed by Xu and Gertner [19], [20], [21] that can take into account correlations among parameters. The improved FAST analysis is implemented using the UASA ToolBox (http://xuchongang.googlepages.com/uasatoolbox) developed by Xu et al. [32].

To statistically compare the importance of different model parameters, standard errors of parametric uncertainty contributions are estimated using a delta method [33]. A sample size of 5000 individual realizations of the model gives us reasonable precision (i.e., small standard errors) for the estimated parametric uncertainty contributions. Uncertainties in the model predictions are measured by variances, which can be greatly affected by any extreme outliers. In order to reduce the effect of those extreme outliers, we exclude simulations where the total number of pupae in the simulated community become larger than 10,000 at any day of the simulation (this occurred in less than 5% of the total number of simulations), which is unrealistic given that the mean and standard deviation of the total number of pupae in our simulated community are about 2,000 and 1,100, respectively, based on the entomological survey. We also observe that, when the population size is larger than 10,000, the population generally keeps increasing through time and does not stabilize, which is not observed in the survey data and is only found in model runs that have a combination of a low level of dependence on food, slow development rate, and low percent of energy used for metabolic activity. In other words, these parameter combinations are unrealistically sampled by the FAST procedure.

Skeeter Buster includes two types of stochasticity: environmental stochasticity and demographic stochasticity. Here, environmental stochasticity mainly refers to stochasticity in food input dynamics, while demographic stochasticity mainly refers to stochasticity in mosquito development, survival and dispersal. In order to understand the importance of stochastic uncertainty, we quantify it by carrying out a second model run for each of the 5000 parameter sets sampled by FAST and examining differences in predicted population densities between pairs of model runs (see Text S4 for technical details). This involves a total of 10,000 simulations. In total, using five desktop computers (Intel Xeon class CPU running at 2.8 GHz), it takes about two weeks to complete the described FAST analysis for this model.

## Results

**Figure 1:**
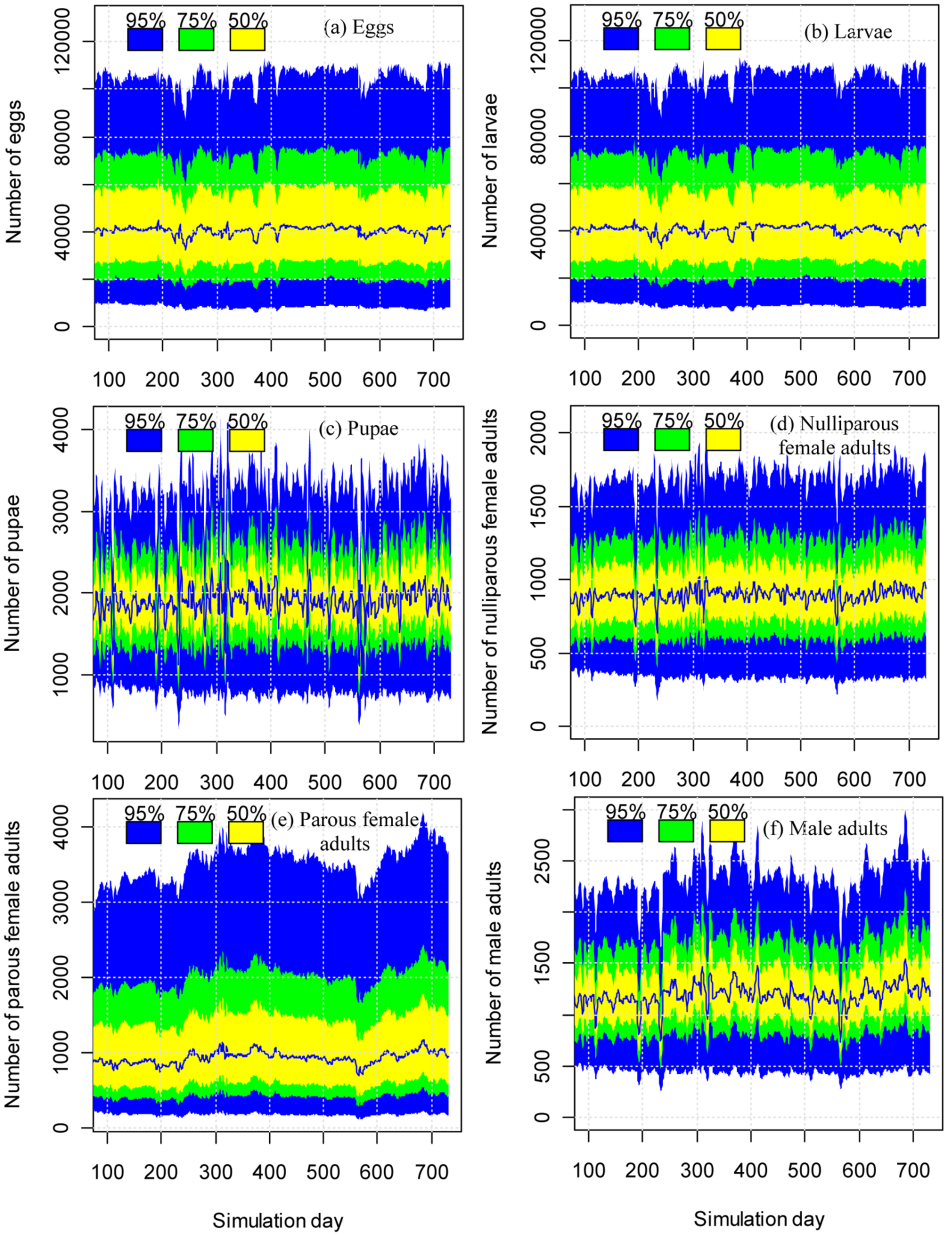
Uncertainties in the predicted population density for different life stages at the community level. In each panel, the central line represents the median of predicted population density based on outputs of simulations carried out using 5000 parameter sets sampled by FAST. The yellow, green, blue and grey bands represent the 50%, 75% and 95% confidence interval of the prediction, respectively.

FAST analysis shows that the median predicted pupal density at the community level (i.e., the total number of pupae in the 612 houses simulated) is around 2000 (Figure 1 c, in which the median is based on 5000 simulations using parameter sets sampled by FAST). This is equivalent to about 3.27 pupae per house, close to the average of 3.54 pupae per house in the survey data [25]. The median predicted population density of female adults at the community level is about 1900 (Figure 1 d and e) and the median population density of male adults is about 1200 at the community level (Figure 1 f), resulting in a median of about 5 adults per house. The median population density of male adults is about two thirds of that of female adults due to the lower survival rate of male adults (see Table S1). There are more adults than pupae because the adult stage lasts longer than the pupal stage.

### Uncertainty in population density at the community level

Our results show that with the inclusion of uncertainties in biological parameters and stochastic uncertainty resulting from environmental and demographic stochasticity, the Skeeter Buster model provides community-level predictions of mosquito population density within a reasonable range (Figure 1). The 95% confidence interval of the population density at the community level of 612 houses ranges from about 20,000 to 100,000 for both eggs and larvae (Figure 1 a,b), from 1000 to 3000 for pupae (Figure 1c), from 400 to 1700 for nulliparous female adults (Figure 1 d), from 300 to 3200 for parous female adults (Figure 1 e), and from 500 to 2200 for male adults (Figure 1f). Levels of uncertainty remain roughly constant over time as a result of constrained food inputs.

**Figure 2:**
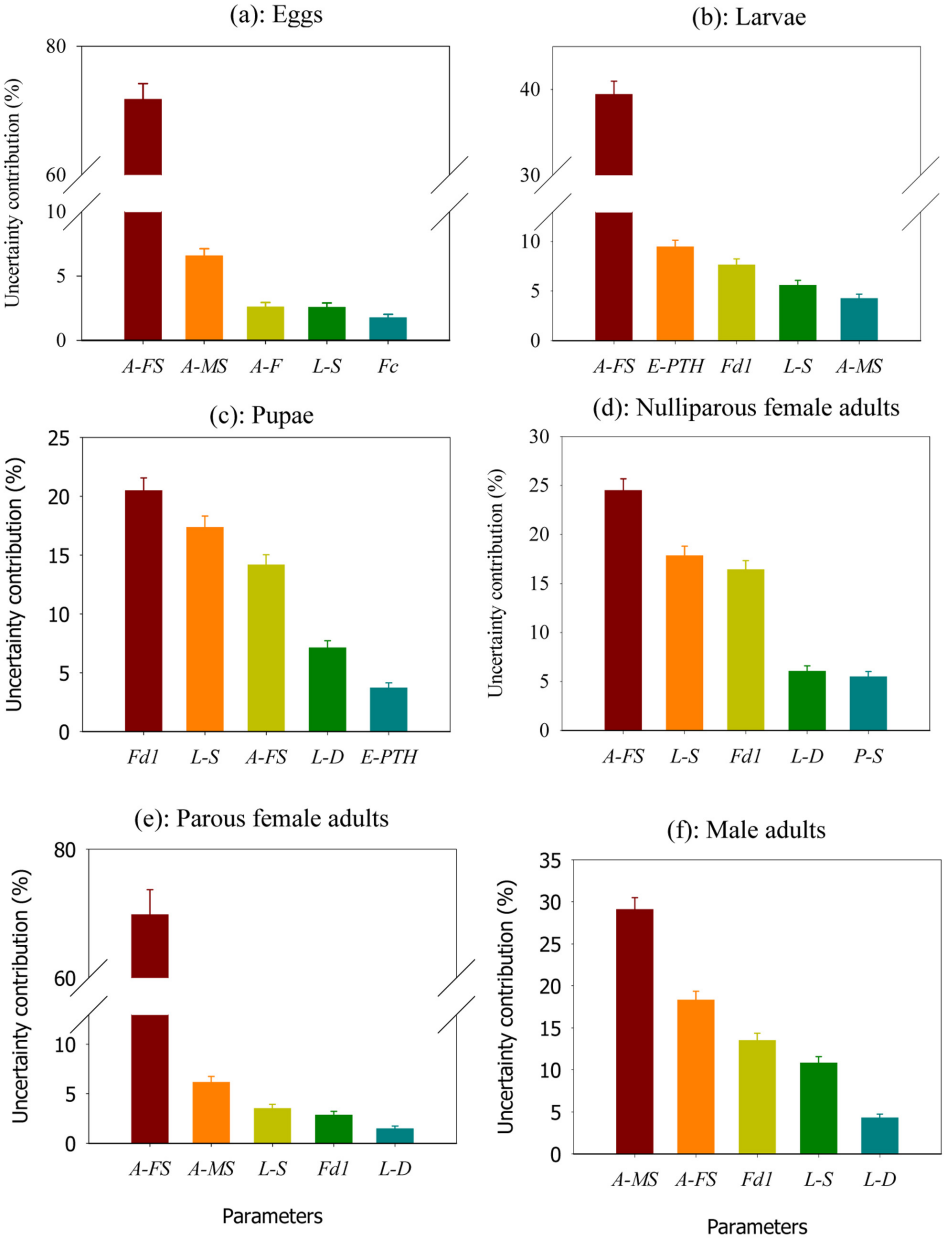
Contributions by different model parameters to uncertainty in the predicted community-level population density at different life stages. Uncertainty analysis is carried out on average population densities predicted over the second simulation year. The vertical bars represent standard errors (plot shows mean +/− standard error). To simplify this figure, we only plot the five parameters that contribute most to the uncertainty in each case. Please see Table S6, S7, S8, S9, S10, S11 for a complete list. A-FS: nominal survival rate for female adults; A-MS: nominal survival rate for male adults; A-F: coefficient of fecundity for female adults; E-PTH: high temperature limit for predator activities on eggs (°C); E-SPTH: survival factor of predation at high temperatures for eggs; Fc: coefficient of food dependence for larvae; Fd1: coefficient of metabolic weight loss for larvae; L-D: larval development rate; L-S: nominal survival rate for larvae. See Table 1 for a detailed explanation of each parameter.

**Figure 3:**
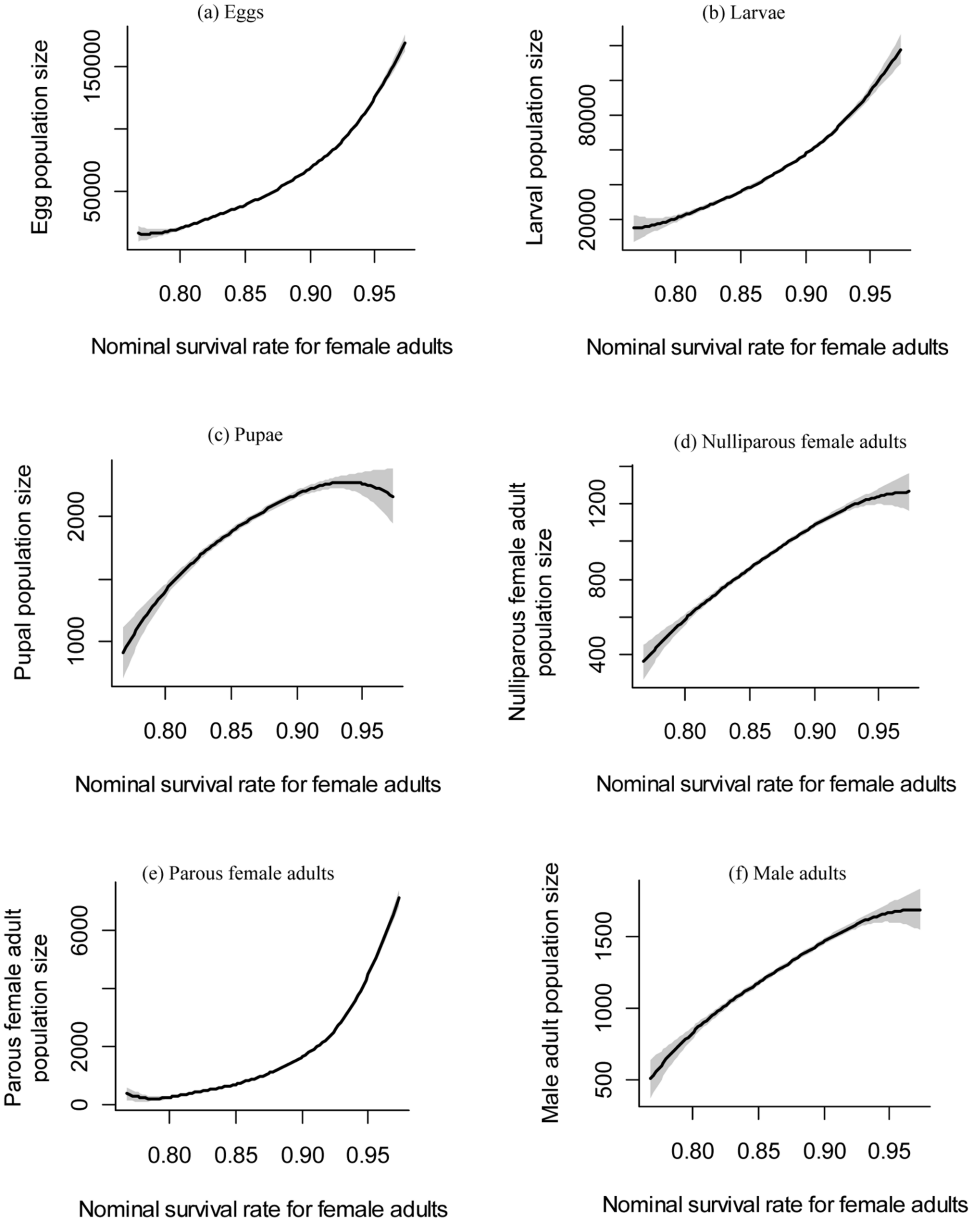
Dependence of community-level population densities at different life stages on the nominal survival rate for female adults. The population density is averaged over the second simulation year. Cubic smoothing spline curves (fitted using the SemiPar R package [45]) show the relationship between the parameter values sampled by FAST and the resulting population density predictions. The shaded areas are the 95% confidence intervals of fitted curves.

For important parameters contributing to the uncertainty in predicted population density averaged over the second simulation year at each life stage, please see Figure 2 and Tables S6, S7, S8, S9, S10, S11. Generally, uncertainty in model parameters explains about 80% or more of the uncertainty in the model predictions. The uncertainty not explained by the main effects includes two components: 1) interactions among parameters; and 2) environmental and demographic stochasticity simulated in the model resulting from natural and individual variability. Of all the parameters in the model, four stand out as very important for most life stages. They are the nominal survival rate for female adults and for larvae, the coefficient of metabolic weight loss, and the larval development rate. The nominal survival rate for female adults accounts for about 72%, 70%, 40%, 24%, 18% and 14% of uncertainty in the predicted egg density, parous female adult density, larval density, nulliparous female adult density, male adult density, and pupal density, respectively. There are relatively strong nonlinear effects of nominal female adult survival rate on the predicted population density of parous female adults, egg and larvae (Figure 3). The strong nonlinear effect of female adult survival rate on parous female adult density results from the fact that this daily survival rate is multiplied repeatedly throughout the life stage of parous female adults. Therefore a large value can have a much stronger effect on parous female adult density than a small value of this survival rate. Given that egg and larval population densities are mainly determined by the density of parous female adults, both of these densities also experience a strong nonlinear dependence on the female adult survival rate (Figure 2).

The coefficient of metabolic weight loss accounts for 21%, 16%, 14%, 8%, and 3% of uncertainty in the predicted pupal density, nulliparous female adult density, male adult density, larval density, and parous female adult density, respectively. The coefficient of metabolic weight loss is important for two reasons. First, when metabolic weight loss is high, less of the energy obtained from consuming food is available for larval growth, which could result in smaller larval body sizes and a smaller number of mosquitoes given the same amount of food (See Figure S5 for a detailed illustration of the effect of coefficient of metabolic weight loss on predicted population size). Second, a large metabolic weight loss can result in a relatively long larval development time as is dependent on larval weight, leading to a lower overall survival rate at the larval stage and a reduced number of mosquitoes. For larvae and parous female adults , because the nominal survival rate of female adults has more dominant effects, the coefficient of metabolic weight loss become less important.

The nominal survival rate of larvae accounts for 18%, 17%, 12%, 6%, 3%, and 2% of uncertainty in the prediction of nulliparous female adult density, pupal density, male adult density, larval density, parous female adult density, and egg density, respectively. The nominal survival rate of larvae is an important factor determining the outcome of development from eggs to adults as a result of the relatively long development time of larvae. For parous female adult and egg density, nominal survival rate of larvae becomes less important since they are less dependent on the larval stage. The larval development rate explains about 7%, 6%, 4% and 2% of uncertainty in the prediction of pupal density, nulliparous female adult density, male adult density, and parous female adult density, respectively. The development rate is important because it can affect the duration of larval stage, which can affect the overall larval survival (a longer larval development time may lead to a lower overall survival rate at the larval stage , given a fixed rate of daily survival probability).

Our results also show that parameters of predator activities for eggs (high temperature limit of predator activities and the survival factor of predation at high temperatures) are very important sources of uncertainty in the predicted population density at the larval stage (Figure 2 b), but are not so important for other life stages. This is because egg survival only affects the early larval stage. For the late larval, pupal and adult life stages, other limiting factors are more important (e.g., coefficient of metabolic weight loss, larval and female adult survival rate).

**Figure 4:**
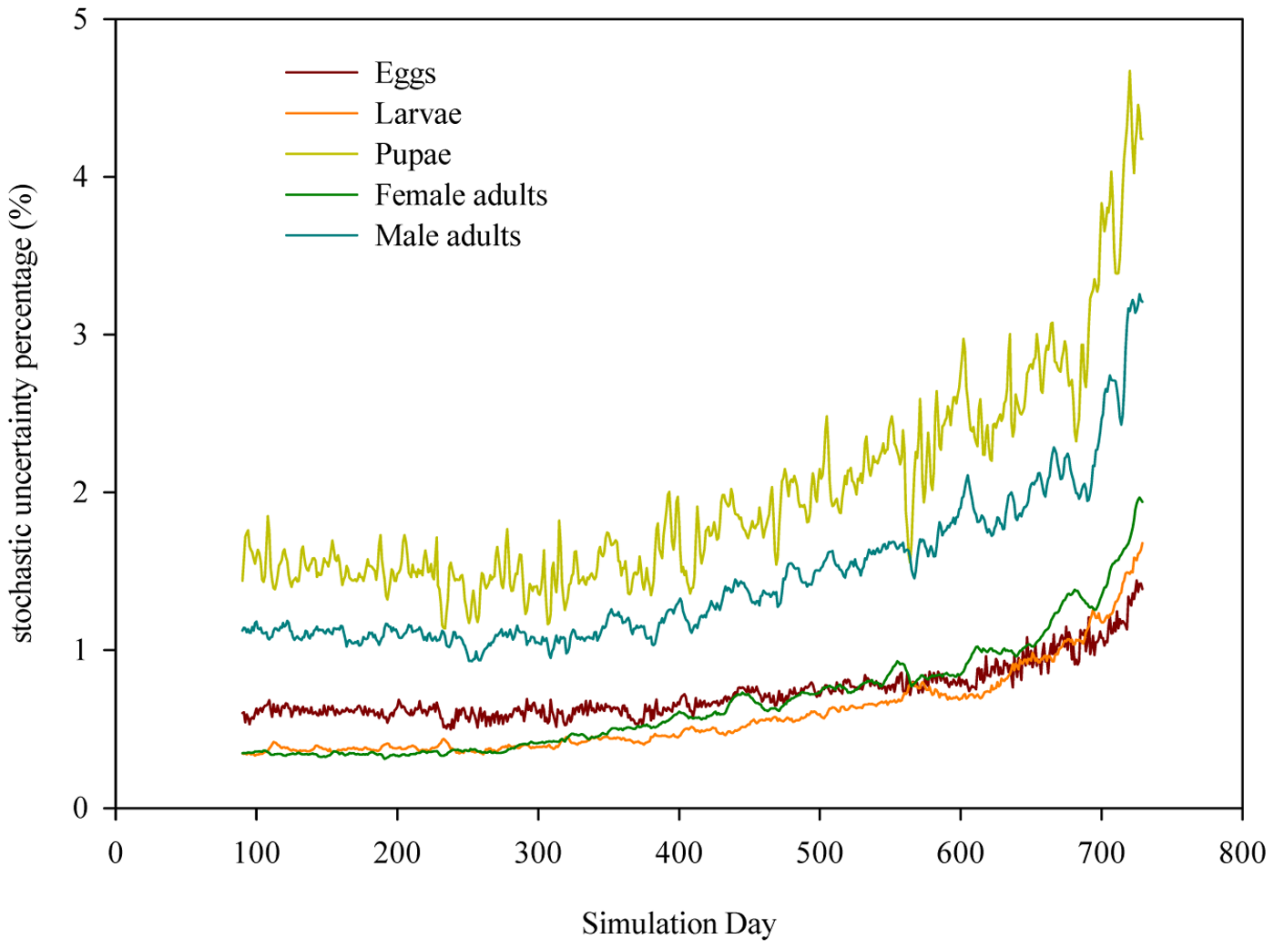
The percentage of uncertainty in the predicted population density contributed by environmental and demographic stochasticity on each simulation day. The environmental stochasticity mainly refers to stochasticity in food input dynamics, while demographic stochasticity mainly refers to stochasticity in mosquito development, survival and dispersal. Stochastic uncertainty is estimated based on the difference in predicted population densities between two replicates of model runs on parameter sets sampled by FAST (a total of 10,000 simulations, see Text S4 for technical details).

Our results show that for each life stage, stochastic uncertainty accounts for less than 5% of uncertainty in the predicted community-level population density on each day throughout the two-year simulation period (Figure 4). This suggests that stochastic uncertainty is relatively low compared to parametric uncertainty for community-level population dynamics. The stochastic uncertainty increases slightly through simulation time due to the accumulation of stochasticity in food input dynamics, dispersal, development and survival. The stochastic uncertainty contribution is relatively higher for pupae and male adults compared to other life stages, largely as a consequence of their smaller population sizes leaving them more prone to stochastic environmental perturbations (e.g., low temperatures).

### Uncertainty in predicted population density at the individual-house level

In this section, we quantify uncertainty in the predicted population densities for each individual house at a time close to the end of simulation period (simulation day 720). Means, standard deviations and coefficients of variation (CV) of population density are calculated for each individual house to measure the spatial uncertainty, based on 5,000 simulations using the parameter sets sampled by FAST. Proportions of uncertainty in the predicted population density at each individual house contributed by different parameters are estimated using FAST, and the proportion of uncertainty contributed by stochasticity is estimated using two replicates of the FAST sample (see Text S4).

**Figure 5:**
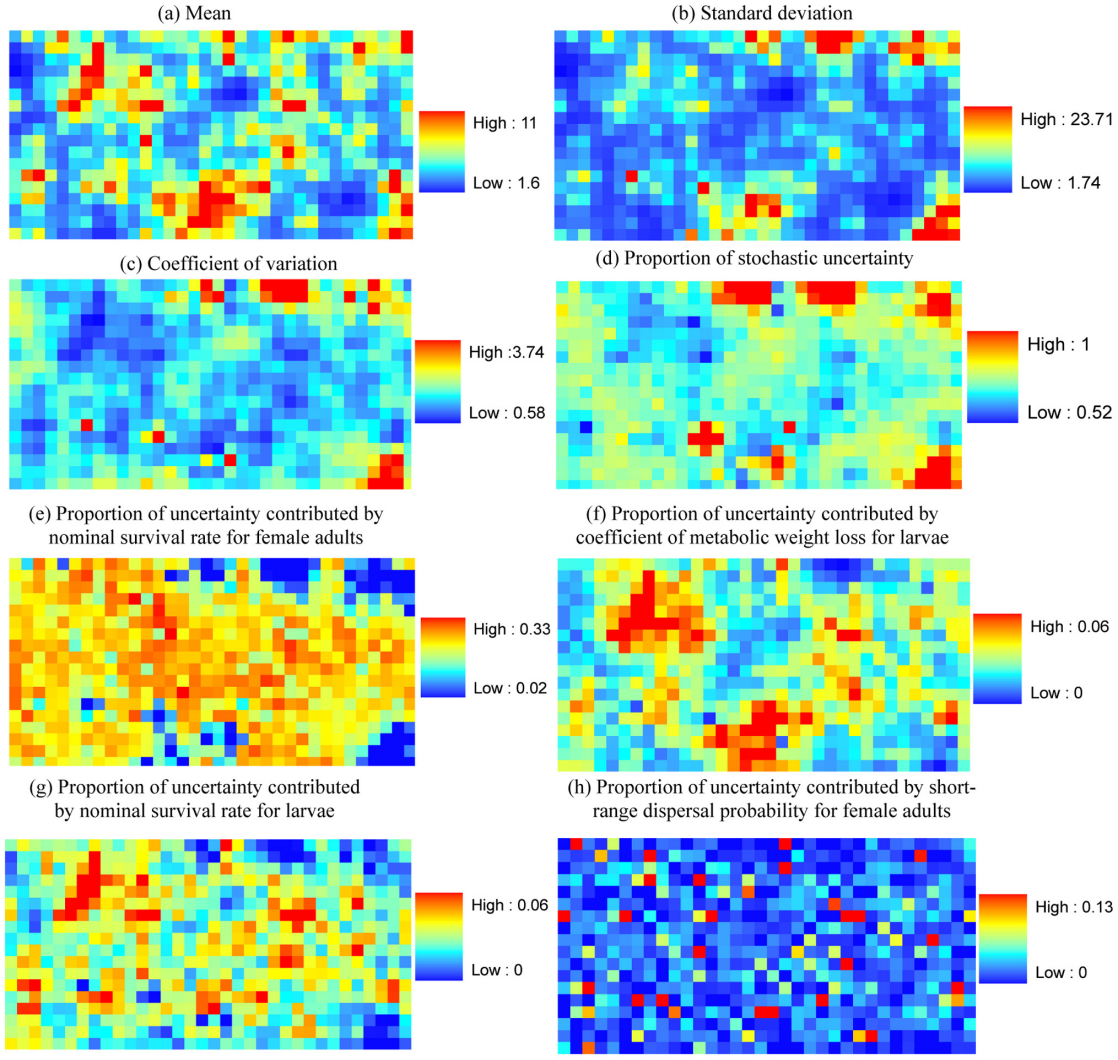
Uncertainty in the predicted female adult population density at the individual-house level on simulation day 720. For each individual house, we quantify uncertainty in the predicted population density (as is described by the (a) mean, (b) standard deviation, and (c) coefficient of variation of predicted population density across the parameter sets sampled by FAST), (d) the proportion of uncertainty contributed by stochasticity, and (e–h) the proportions of uncertainty contributed by specific model parameters. To simplify this figure, only parameters with uncertainty contributions in any house larger than 5% are plotted.

Our results show that the standard deviation of predicted mosquito population density for each life stage is low for houses where the mean population density is relatively low (the main text only presents results for the female adult population distribution, see Figure 5 a, b; see Figure S6, S7, S8, S9 for male adult, egg, larval and pupal distributions). However, the corresponding coefficient of variation and the proportion of stochastic uncertainty are much higher (Figure 5 c, d and Figure S6, S7, S8, S9 c, d). It is noticeable that stochasticity explains more than 50% of uncertainty in the predicted population density in every house for all life stages except for larvae. For the larval density prediction, the proportion of stochastic uncertainty is high (>40%) for most of the houses, except for a few houses with relatively large food inputs (Figure S8). The proportion of stochastic uncertainty at the individual-house level is substantially higher than that at the community level at the same simulation day (<5%) (see Figure 4).

In terms of parametric contributions to uncertainty in the predicted population density, the nominal survival rate for female adults is important for all houses except for a few houses where the proportion of stochastic uncertainty is very high (Figure 5 e and Figure S6, S7, S8, S9 e). The coefficient of metabolic weight loss and larval survival rate are more important where there is a relatively larger amount of food inputs either in the house, or in neighboring houses (Figure 5 f, g and Figure S6, S7, S8, S9). This is because relatively larger food inputs can lead to a higher population density so that the coefficient of metabolic weight loss and larval survival rate can have more important effects on local larval and pupal population density. Our results show that spatial dispersal is much more important for population densities in those few houses where the food inputs are large (Figure 5 h and Figure S6, S7, S8, S9) compared to other houses with small food inputs. The main reason for this is that a high dispersal rate will result in a large number of mosquitoes spreading out from these houses. For houses with small food inputs, dispersal may still contribute to the population dynamics (due to the in-flow of dispersing mosquitoes from houses with relatively large food inputs) but to a lesser extent as a result of stochasticity in dispersal. The effect of short-range dispersal on population density is much weaker for pupae (See Figure S9 h), which depends more on the amount of food held by water containers in and around the house.

**Figure 6:**
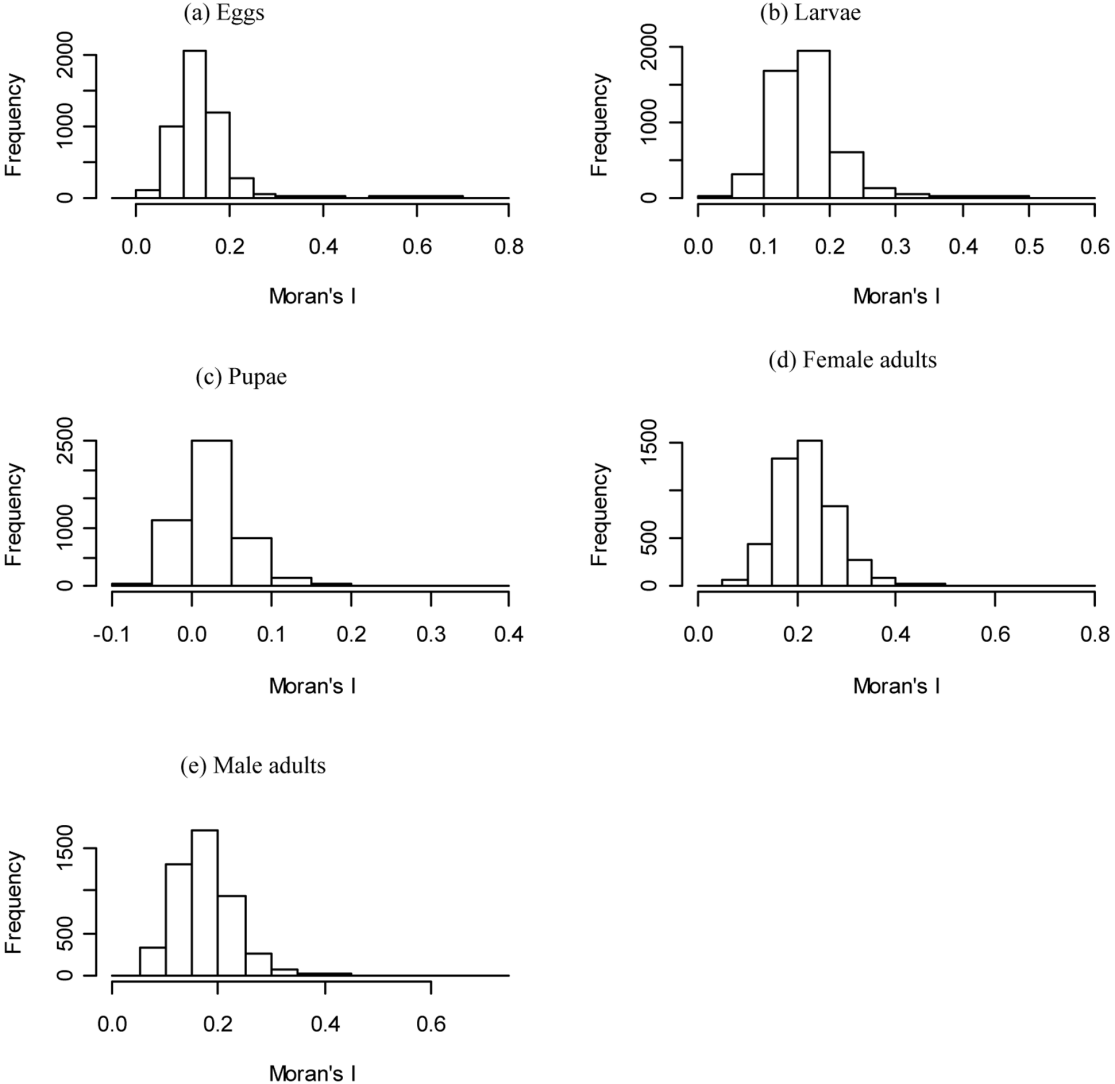
Histograms of Moran's *I* for the population distribution at simulation day 720. A Moran's *I* near +1.0 indicates clustering; an index value near −1.0 indicates dispersion; and an index of 0 indicates complete randomness [46]. See Text S5 for details of the calculation of Moran's *I*.

Our results show that distributions of female and male adults are spatially clustered (Figure 5a and Figure S6 a). The clustering of egg distribution is similar to that of female adults (Figure 5a and Figure S7 a), while larvae and pupae are less clustered (Figure S8 a and Figure S9 a). This is because larvae and pupae are more dependent on the water containers and the amount of food they hold, neither of which is clustered in the model input. Based on a spatial statistic of Moran's *I* (see Text S5) calculated for each individual simulation (Figure 6), we show that there is no significant spatial clustering for pupae (the p-values for Moran's *I* are not shown but are mostly larger than 0.05), while there is some degree of spatial clustering for other life stages.

**Figure 7:**
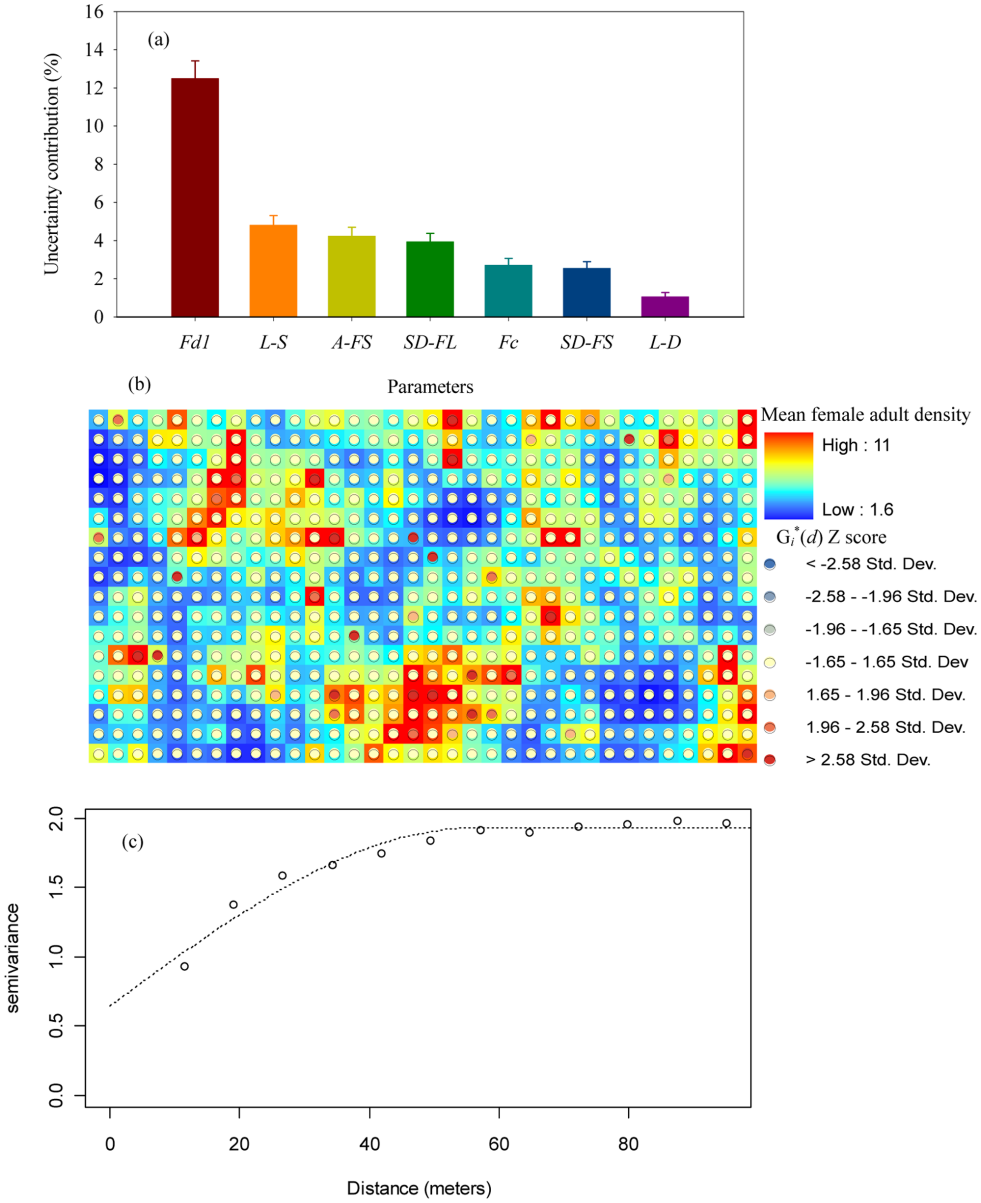
Drivers for the spatial clustering pattern as measured by Moran's *I*. a) proportions of uncertainty in Moran's *I* contributed by different model parameters; b) overlay of the hot spots of food input distribution (as identified by the Getis G_i_*(*d*) score with inverse distance weights) with distribution map of mean female adult density at individual houses (based on model simulations with parameter sets sampled by FAST); and c) the semivariogram of mean population density distribution for female adults. The semivariogram describes the spatial dependence in population density at the individual-house level, with higher values indicating lower spatial autocorrelation. See Text S5 for details of the calculations of both Getis G_i_*(*d*) score and semivariogram. A-FS: nominal survival rate for female adults; Fc: coefficient of food dependence for larvae; Fd1: coefficient of metabolic weight loss for larvae; L-D: larval development rate; L-S: nominal survival rate for larvae; SD-FL: Long-range dispersal probability for female adults; SD-FS: Short-range dispersal probability for female adults. See Table 1 for a detailed explanation of each parameter.

Applying FAST analysis to the level of spatial clustering of female adults as measured by Moran's *I*, our results show that the most important factor affecting spatial clustering is the coefficient of metabolic weight loss (Figure 7 a). Other important parameters include the nominal survival rate for larvae and for female adults, the short-range and long-range dispersal probabilities for female adults, and the coefficient of food dependence [a coefficient specifying the effect of food inputs in water-containers on larval body weight gain, with a lower value indicating a stronger effect of food on larval growth and a higher level of density dependence (see Text S2 for more explanations)]. If we superimpose the hot spots of houses with large food inputs as identified by a G_i_^*^(*d*) statistic [34], [35] (see Text S5) onto the female adult population density map (Figure 7 b), we can see that high female adult population densities generally occur at or near houses with large food inputs. This suggests that high local population density (determined by food inputs, survival rate, coefficient of metabolic weight loss, and coefficient of food dependence) and spatial dispersal (determined by mosquito longevity and dispersal probability) are both important for forming the spatial clustering pattern as measured by the Moran's *I* statistic. If we calculate the semi-variance of female adult distribution (a statistic to measure the strength of spatial autocorrelation, see Text S5 for details, using the spatial distribution of mean population densities at each individual house which are based on parameter sets sampled by FAST), we can show that the semi-variance stabilizes at a distance of 40–50 meters (or, equivalently, 4–5 houses) (Figure 7 c). This suggests that, even though the spatial distribution of food input is not clustered at the level of individual houses, the distribution of adult mosquitoes may have clustering patterns if houses with large amount of food inputs are within a distance of 4–5 houses. This distance is close to that obtained in a previous empirical study indicating that the mosquito data for Iquitos exhibits a weak spatial clustering of *Ae. aegypti* at a distance of 30 meters [26].

### Temporal variability of population density

To gain a better understanding of the population dynamics of *Ae. aegypti*, we also examine factors contributing to the temporal variability at the community and the individual house level. Temporal variability may result from stochastic uncertainty, biological development cycles, environmental factors (e.g., temperature) and temporal dynamics of food in water-filled containers. For the temporal variability of population dynamics at the community level, our results show that important parameters include the temperature limits for survival and predation of eggs, the gonotrophic development rate, and the nominal survival rate for female adults (See Text S6). Additionally, at the individual-house level, the spatial dispersal of adult mosquitoes and the coefficient of food dependence are also important parameters (see Text S7).

## Discussion

Our results show that uncertainty in the estimate of nominal survival rate for female adults is the most important source of uncertainty for the prediction of population densities of all life stages by Skeeter Buster at both community and individual-house levels. Thus, it is important that researchers develop more accurate and precise empirical estimates of this parameter. Current estimates of survival are mainly based on mark-release-recapture methods, which may be subject to a number of errors (e.g., sampling errors, spatial heterogeneity, and environmental stochasticity). Thus, it could be difficult to reduce uncertainty in the estimate of adult survival. Mosquito adult survival can be affected by both intrinsic biological factors (e.g., age-related internal physiological deterioration causing senescence) and extrinsic abiotic and biotic factors (e.g., predation, temperature and moisture). An important source of uncertainty for the estimation of adult survival rate is due to the uncertainty in predation. Improved model predictions may require a decoupling of the effects of intrinsic and extrinsic factors on mosquito survival.

Our study shows that the coefficient of metabolic weight loss, a parameter describing food utilization by larvae, is important for uncertainty in the predicted mosquito population density. In view of the potentially large amount of uncertainty in the estimation of food inputs—an uncertainty which is not explored in our current study—future research on food quantification and food limitation for larval development should provide a better understanding of population dynamics. However, in the field, the effect of food on larval growth and productivity can be very different depending on the leaf species [36], nutrient content [37], [38], algae abundance [39], and microbial community [40]. Thus, it would be difficult to directly measure the amount of food available for larval growth. The weight gain model used both in Skeeter Buster and CIMSiM is mainly based on the laboratory work of Gilpin and McClelland [3] using liver powder as the food source for larvae. This may not be realistic, but at least provides us a way to estimate the amount of food (equivalent to liver powder) available for larval growth by fitting the model to field survey data [24]. However, food inputs for the weight gain model could be a very important source of uncertainty in Skeeter Buster. Currently we are conducting field experiments in Mexico to explore density dependent effects on larval growth and survival [41], which may improve the weight gain model in the future.

Our results show that spatial dispersal importantly affects population density and spatial pattern (as measured by the Moran's *I*) at the individual-house level. However, it does not have an important effect on population density of any life stage at the community level. This is because mosquitoes are present in almost every house of our simulated study area with plentiful availability of containers for mosquito oviposition. Dispersal only balances the population density among individual houses, but does not have much effect on the overall population density at the community level. If severe environmental conditions during the winter in temperate areas or vector control practices lead to a situation in which mosquitoes only survive in a small proportion of containers in refuge sites, then dispersal is likely to be important for the population density during the period of population recovery at the community level [11]. We also notice that dispersal is a particularly important source of uncertainty in the predicted population densities within houses that have the greatest food inputs, due to population outflow by dispersal. This could have important implications for the dynamics of disease spread because dengue infections in houses with high mosquito population density may pose a high risk of disease spread to nearby houses if a large number of infected mosquitoes move to nearby houses.

Our results show that, compared to parametric uncertainty, stochastic variation does not produce substantial uncertainty in predicted population density at the community level. However, at the level of individual houses, stochastic uncertainty accounts for more than 50% of uncertainty in the predicted population density for houses with relatively small food inputs. Because stochastic uncertainty is generally irreducible, it could be very difficult to improve the precision of mosquito population density in individual houses even if we could substantially reduce parametric uncertainty in the future. Although stochastic uncertainty is high at the individual-house level, our results indicate that the spatial clustering pattern as measured by Moran's *I* is jointly determined by the food input, the food utilization by mosquitoes, the spatial dispersal of adult mosquitoes, and their longevity as determined by the survival rate. This suggests that the spatial model can be used to predict the spatial clustering of population density at the individual-house level given the spatial distribution of containers.

Uncertainty in the model structure and in model data inputs (e.g., container data) can both be important sources of uncertainty. We did not quantify those uncertainties in this study mainly due to the lack of currently available information. One example of structural uncertainty is in the water temperature calculations. The Skeeter Buster model uses a polynomial regression to calculate water temperature using air temperature and container shading based on data from Florida. An alternative approach has been provided by Kearney et al. [42] who coupled transient-state energy and mass balance equations to calculate daily temperature cycles in containers differing in size, catchment and degree of shading. This type of biophysical model of energy and mass transfer could potentially increase the prediction accuracy of water temperatures, which could be important for larval and pupal development.

Uncertainty analysis can be used to characterize the importance of uncertainties that accompany the use of complex models. Quantification of uncertainty provides an indication of the reliability of predictions made by the model, making uncertainty analysis an indispensible step in the deployment of complex models. Our uncertainty analysis has identified parameters whose uncertainties have an important impact on the predictive ability of the model. Future studies should attempt to improve the estimates of these parameters, which will likely require the collection of additional data and reanalysis of existing data. Our reliance on expert knowledge to quantify the uncertainties of individual parameters means that the results of our uncertainty analysis are, to some extent, impacted by the biases to which the process of elicitation of expert opinion are prone [43].However, Bayesian data analysis techniques [44] can be used to reduce such biases and improve estimates of parameters, by combining prior information (prior distributions for each parameter, informed by expert opinion) with information drawn from appropriate experimental and observational data.

Although the uncertainty analysis results in this paper are based on the application of Skeeter Buster model to the Peruvian city of Iquitos, many of the results are likely to hold if the model were applied to other tropical areas. The insight gained into the importance of specific model parameters can provide general directions for the future improvement of models for mosquito population dynamics.

## Supporting Information

Text S1 Parametric uncertainty quantification.
(0.23 MB DOC)

Text S2 Parameter estimation for the larval weight gain model.
(0.09 MB DOC)

Text S3 Parameter estimation for the enzyme kinetics model.
(0.07 MB DOC)

Text S4 Quantification of stochastic uncertainty.
(0.04 MB DOC)

Text S5 Spatial statistics.
(0.04 MB DOC)

Text S6 Temporal variability of population density at the community level.
(0.06 MB DOC)

Text S7 Temporal variability of population density at the individual-house level.
(0.63 MB DOC)

Figure S1 Survival factor as a function of temperature. The survival factor ranges between 0 and 1 and is multiplied with nominal survival rate to get the temperature-dependent survival rate. T_min_ is the minimum temperature for survival, below which the low temperature has a strong effect on mosquito survival (the survival factor is generally less than 0.05); T_low_ is the low temperature limit below which is suboptimal for mosquito survival; T_high_ is the high temperature limit above which is suboptimal for mosquito surivival; T_max_ is the maximum temperature for survival, above which the high temperature has a very strong effect on mosquito survival (the survival factor is generally less than 0.05).
(0.07 MB TIF)

Figure S2 Survival factor as a function of saturation deficit (SD). The survival factor ranges between 0 and 1 and is multiplied with nominal survival rate to get the humidity-dependent survival rate. SD_low_ is the low saturation deficit limit below which saturation deficit has little effect on mosquito survival. The survival rate decreases linearly between SD_low_ and SD_high_, the high saturation deficit limit above which the saturation deficit has a strong effect on mosquito survival (survival factor is low).
(0.06 MB TIF)

Figure S3 Histograms of air and water temperatures (degrees Celsius) in Iquitos for year 2000. The container water temperatures are simulated using a polynomial function obtained from a regression of water temperature on air temperature and sun exposure for 12 containers monitored for 76 days in Gainesville, FL, USA [4]. The water temperature is calculated assuming a sun exposure of 0.5 for the container.
(0.49 MB TIF)

Figure S4 Sum of daily food input from different containers (Unit: mg/day) at individual houses. Each block/cell represents a single house. The food inputs are fitted to the pupal data in the mosquito survey at individual houses in Iquitos [23]. The food inputs are not spatially clustered based on the Moran's I statistic [46] using inverse distance weights (I = 0.005, p-value = 0.82).
(0.21 MB TIF)

Figure S5 Dependence of community-level population density on coefficient of metabolic weight loss at different life stages. The curves are fitted to the scatter plot of parameter values sampled by FAST and the corresponding predicted population densities using cubic smoothing splines with the SemiPar R package [45]. The shaded areas are the 95% confidence intervals of the fitted lines.
(0.58 MB TIF)

Figure S6 Uncertainty in the predicted male adult population density at the individual-house level on simulation day 720. For each individual house, we quantify uncertainty in the predicted population density (as is jointly described by the (a) mean, (b) standard deviation, and (c) coefficient of variation of predicted population density across the parameter sets sampled by FAST), (d) the proportion of uncertainty contributed by stochasticity, and (e–i) the proportions of uncertainty contributed by specific model parameters. To simplify this figure, only parameters with uncertainty contributions in any house larger than 5% are plotted.
(1.47 MB TIF)

Figure S7 Uncertainty in the predicted egg density at the individual-house level on simulation day 720. For each individual house, we quantify uncertainty in the predicted population density (as is jointly described by the (a) mean, (b) standard deviation, and (c) coefficient of variation of predicted population density across the parameter sets sampled by FAST), (d) the proportion of uncertainty contributed by stochasticity, and (e–g) the proportions of uncertainty contributed by specific model parameters. To simplify this figure, only parameters with uncertainty contributions in any house larger than 5% are plotted.
(1.75 MB TIF)

Figure S8 Uncertainty in the predicted larval population density at the individual-house level on simulation day 720. For each individual house, we quantify uncertainty in the population density (as is jointly described by the (a) mean, (b) standard deviation, and (c) coefficient of variation of predicted population density across the parameter sets sampled by FAST), (d) the proportion of uncertainty contributed by stochasticity, and (e–g) the proportions of uncertainty contributed by specific model parameters. To simplify this figure, only parameters with uncertainty contributions in any house larger than 5% are plotted.
(1.67 MB TIF)

Figure S9 Uncertainty in the predicted pupal density at the individual-house level on simulation day 720. For each individual house, we quantify uncertainty in the predicted population density (as is jointly described by the (a) mean, (b) standard deviation, and (c) coefficient of variation of predicted population density across the parameter sets sampled by FAST), (d) the proportion of uncertainty contributed by stochasticity, and (e–h) the proportions of uncertainty contributed by specific model parameters. To simplify this figure, only parameters with maximum uncertainty contributions larger than 5% in any house are plotted except for panel (h), which is shown for the comparison of mosquito dispersal importance at different life stages.
(1.67 MB TIF)

Table S1 Uncertainties in the estimates of parameters for adults.
(0.11 MB DOC)

Table S2 Uncertainties in the estimates of parameters for larvae and pupae.
(0.09 MB DOC)

Table S3 Uncertainties in the estimates of parameters for egg survival and hatching.
(0.09 MB DOC)

Table S4 Uncertainties in the estimates of parameters for larval weight gain.
(0.06 MB DOC)

Table S5 Uncertainties in the estimates of parameters for mosquito dispersal.
(0.07 MB DOC)

Table S6 Uncertainty contributions (%) by different model parameters for predicted egg population density at the community level.
(0.05 MB DOC)

Table S7 Uncertainty contributions (%) by different model parameters for predicted larval population density at the community level.
(0.05 MB DOC)

Table S8 Uncertainty contributions (%) by different model parameters for predicted pupal population density at the community level.
(0.05 MB DOC)

Table S9 Uncertainty contributions (%) by different model parameters for the predicted population density of nulliparous female adults at the community level.
(0.05 MB DOC)

Table S10 Uncertainty contributions (%) by different model parameters for the predicted population density of parous female adults at the community level.
(0.04 MB DOC)

Table S11 Uncertainty contributions (%) by different model parameters for the predicted population density of male adults at the community level.
(0.05 MB DOC)
