# Supplementary material for: Understanding Uncertainties in Model-Based Predictions of Aedes aegypti Population Dynamics
Source: PLoS Negl Trop Dis. 2010 Sep 28;4(9):e830. doi: 10.1371/journal.pntd.0000830 (PMC2946899; doi:10.1371/journal.pntd.0000830)
Supplement: Text S1 — Parametric uncertainty quantification. (0.23 MB DOC) [file pntd.0000830.s001.doc]

# Text S1: Parametric uncertainty quantification

We gauged the level of uncertainty related to values of each parameter based on both literature and expert knowledge. For use of expert knowledge, we conducted workshops in 2008 and 2009 that included members of our own lab and two other mosquito ecology labs: Professor Thomas Scott’s Lab (University of California, Davis) and Professor Laura Harrington’s Lab (Cornell University). In the workshops, we explained the meaning of each parameter to the participants and we described to them our approach for visualizing and quantifying values of uncertainty (see Figure S1.1).

Frequency

No confidence

Low confidence

Moderate confidence

High confidence

Parameter values

Parameter values

Parameter values

Parameter values

Figure S1.1 Probability distributions used to describe differing levels of uncertainty in the value of a parameter, *x*, whose default value is *x*_default_ and for which the lower and upper limits are *x*_min_ and *x*_max_. Uncertainty in the value of *x* is described by a beta distribution, with the scaled value *y* = (*x* – *x*_min_)/( *x*_max_ - *x*_min_) following a beta distribution with parameters α and β. We take α = γ (*x*_default_ – *x*_min_)/( *x*_max_ - *x*_min_) and β = γ (*x*_max_ – *x*_default_)/( *x*_max_ - *x*_min_). The parameter γ characterizes the confidence in the default value of the parameter, with γ = 4 if confidence is low, γ = 10 if confidence is moderate, and γ = 20 if confidence is high. For the examples shown, we have assumed that *x*_default_ falls exactly halfway between the lower and upper limits, i.e. *x*_default_ = ( *x*_min +_ *x*_max_ )/2, giving symmetric distributions. In general, however, the distributions are asymmetric about *x*_default_.

The participants were asked to provide, for each parameter, their expert opinion on the extreme possible values the parameter could take and their confidence in the default value of this parameter (the assumed most likely value based on data and experience), expressed as one of four levels of confidence: 1) no confidence; 2) low confidence; 3) moderate confidence; and 4) high confidence (see Figure S1.1). We emphasized to the participants that we wanted their answers to reflect, as much as possible, the views of the mosquito ecology research community. We then defined the range of possible values for each parameter based on the collected opinions on extreme values, and we used the expressed level of confidence to define the probability distribution of specific values within this range. If we had no confidence for a particular default value, then we assigned a uniform distribution to the probability distribution over the possible range. Otherwise, we used a beta distribution, scaled to the possible range, to assign higher probabilities to values near the default value, setting the likelihood of the default value in the low, moderate and high confidence scenarios to be approximately 1.5, 2.5 and 3.5 times as large as that defined in the uniform distribution (Figure S1.1). Generally, experts in the workshops agreed on the ranges and confidence levels in the default value for each parameter. When there was no consensus, we used the minimum value provided for the lower range and maximum value provided for the upper range. For the confidence levels in default values, we used the levels with majority agreements.

For parameters not directly measurable in the field or in the lab, it was difficult for the experts to provide their knowledge about the uncertainty. For most parameters, the literature provides both means (default values) and estimates of experimental uncertainty (e.g., standard errors). However, for parameters in the weight gain model and in the development rate model (see Text S2 and S3), where the original paper only reported the estimated values but no associated uncertainty, we digitized figures in the original paper and re-estimated the parameters to get their possible ranges and the confidence levels of default values (see Text S1.2 and S1.3).

# Uncertainty in survivorship

In Skeeter Buster, adult mosquito survivorship can either be assumed to be age-independent or age-dependent. In this uncertainty analysis, we assume that the survival rate is constant. Since the effect of age-dependent survival for female adults only becomes evident after about 20 days in laboratory experiments [1], this assumption should not have a major effect on the validity of our uncertainty analysis results given the shorter life-spans of mosquitoes in the field resulting from predation and other environmental factors. Based on our workshops and the literature [2,3,4,5,6], the default value for nominal survival rate for female adults and male adults is set at 0.89 and 0.77 respectively. Uncertainty in the nominal survival rate is assigned with a range from 0.75 to 0.99 and a range from 0.72 to 0.99 for female adults and male adults, respectively. Based on our workshops, we conclude that there is moderate confidence in the default values. It should be borne in mind, particularly when considering values at the upper ends of these ranges, that Skeeter Buster considers additional effects of temperature and moisture on mosquito survival. See Table S1, S2 and S3 for details on our use of categories of confidence and quantifications for survival parameters of adults, larvae, pupae and eggs.

# Uncertainty in weight gain model

Uncertainties in the parameters of larval weight gain model are defined based on a re-estimation of parameters using the larval weight data from Gilpin & McClelland [7] and a Metropolis-Hastings fitting algorithm [8,9] (see Text S2 for details). Based on the defined uncertainties in model parameters, for a 200ml cup with 40 mg liver powder and 20 larvae (initial weight = 0.001 mg), the 95% confidence interval for predicted weight after 4 days ranges between 0.28 and 0.62 mg (see Figure S1.2). Uncertainty in the predicted weight gain is relatively larger for larvae with higher body weights due to the propagation of uncertainty in the weight gain model.

Figure S1.2 Uncertainty in the predicted larval weights by the weight gain model with parametric uncertainties defined in Table S4. Initially, 40 mg of food and 20 larvae are present. The yellow, green, blue and grey bands represent the 50% ,75% ,95% confidence interval of the prediction, respectively. The grey band represents the output boundary. The central blue line is the median. Symbols depict data points from Gilpin & McClelland [3] (squares: “house strain”, triangles: “bush strain”).

# Uncertainties in development times

Uncertainty in the estimation of development times was calculated based on a re-estimation of four parameters in a enzyme kinetics model [8, see eq. (S7)] using data from the literature (Tun-Lin et al. [9], Rueda et al. [10], Focks et al. [11] and Farnesi et al. [12]) and a Metropolis-Hastings fitting algorithm (see section S3 in Supplementary Materials). We used standard deviations of estimated development times from the literature to construct possible development time ranges at different temperatures. These ranges defined our uncertainty in the development rate response curve as a function of temperature. This uncertainty in the functional curve was of more interest to us than uncertainties in individual parameters of the enzyme kinetics model, since the parameters themselves have limited biological meaning [13]. Instead, the use of this model should be seen as a means to construct a non-linear relationship between development and temperature. The uncertainty in the estimated relationship between temperature and development rate itself was therefore more relevant than the uncertainties associated with each individual parameter.

We represented uncertainty in the response of development rate to temperature with an ensemble of 2000 development rate response curves generated by the Metropolis-Hastings method. A development rate response curve corresponds to a specific value of each of the four parameters in the enzyme kinetics model, and defines the relationship between development rate and temperature. For the uncertainty analysis of the Skeeter Buster model, we draw random samples of development rate response curves from the ensemble (see Text S3 for details) to quantify the amount of uncertainty in model outputs contributed by uncertainty in the estimated relationship between temperature and development rate.

The resulting temperature-dependent uncertainties for development times for eggs, larvae, pupae and gonotrophic cycle durations are shown in Figure S1.3. We can see that uncertainty in development time is relatively high at lower temperatures, which is attributed to three reasons. First, lower temperatures lengthen the time taken to complete the life stage, which gives a higher potential for individual variability to accumulate. Second, the lower survival rate due to very low temperature will result in a reduced number of individuals completing the specific stage, with the resulting smaller sample size leading to lower confidence for the estimated development time. Third, the model structure itself may not be able to capture the development time at extreme environmental conditions. This is one type of structural uncertainty. The large uncertainty at lower temperatures may play a large role in temperate areas, but may not have much effect on model predictions for our study area since the temperature there is generally higher than 20 ^o^C (see Figure S3).

(b) Larvae

(c) Pupae

(d) Gonotrophic cycles

Temperature (Celsius)

Temperature (Celsius)

(a) Egg

Figure S 1.3 Uncertainty in the estimation of development times for (a) eggs, (b) larvae, (c) pupae and (d) gonotrophic cycle duration as functions of temperature. The embryonic development times for eggs are estimated based on data from Farnesi et al. [12]. The development times for larvae and pupae are estimated based on data from Rueda et al. [10] and Tun-Lin et al. [9]. The estimated uncertainty encompasses data from Kamimura et al. [14]. In panels (b) and (c), the circles represent data from Rueda et al. [10] and the squares (shifted to the right by 0.5 ºC to aid visibility) are data from Tun-Lin et al. [9]. The triangle points (shifted to the left by 0.5 ºC to aid visibility) represent the data from Kamimura et al.[14]. The gonotrophic cycle durations are estimated based on data from Focks et al [11]. The vertical lines indicate the standard deviations (+/- one standard deviation) of the development times. The central line represents the median of the prediction based on the parameter sets sampled by FAST. The yellow, green, blue and grey bands represent the 50% ,75% ,95% confidence interval of the prediction, respectively. The grey band represents the output boundary.

# Uncertainty in spatial dispersal

There is a large amount of uncertainty in the estimate of *Ae. aegypti* dispersal [see 15, Chapter 15 for a review]. The maximum dispersal distance ranges from 100 to 800 meters. The spatial dispersal rate is mainly estimated based on the mark-release-recapture approach and the associated uncertainty is assumed to result from different factors including sampling error, recapture rate, mosquito survival, breeding site availability, and environmental heterogeneity. In the Skeeter Buster model, two types of dispersal are used to model mosquito movement: short-range dispersal (dispersal to a neighboring house), and long-range dispersal (dispersal from the original house to any other within a specified maximum dispersal distance). Harrington et al. [16] showed that, in their mark-release-recapture experiments, the majority of mosquitoes (72% of males and 65% of females) were captured in houses adjacent to their outdoor release location during 4-12 capture days. Thus, our assumption in the Skeeter Buster model that short-range dispersal between neighboring houses is the major dispersal mechanism is consistent with these observations. An adult mosquito may, however, make short-range dispersals on more than one day, so its lifetime dispersal could cover several houses [17]. The uncertainty range of daily probability of short-range dispersal is defined between 0.05 to 0.5 with a default value of 0.3, which is fitted using Harrington’s data [16,17]. The daily probability of long-range dispersal is defined between 0 and 0.1 with a default value of 0.02. The confidence for default values of both female and male adults is defined to be low based both on the literature and expert opinion. See Table S5 for details of uncertainty quantifications for all spatial dispersal parameters.

# Correlation among model parameters

Model parameters are often assumed to be independent. However, they may be correlated as a result of common factors (e.g., common environmental factors or factors controlling the different biological parameters). For example, if a specific environmental factor favors survival of female adults, it is highly likely that the survival of male adults will also be favored. This can lead to a correlation between survival rates of male adults and those of female adults. However, if we assume independence of parameters, the sampling of parameters undertaken as part of the uncertainty analysis could generate unrealistic combinations (e.g., the survival rate for male adults is very high while the survival rate for female adults is very low). Thus, it is important to incorporate potential correlations among parameters. In uncertainty analysis, taking correlation into account for linear models (i.e., the model outputs are linearly dependent on model parameters) can often increase the amount of uncertainty in the model’s predictions if positive correlation is assumed (due to the enhanced population dynamics if sampled values of two parameters are both high, assuming parameters are positively correlated with model output) and decrease the amount of uncertainty in the model’s predictions if negative correlation is assumed (due to the balancing of population dynamics by low and high parameter values, assuming parameters are positively correlated with model output). However, for complex models, the effects of correlation on uncertainty can be different due to the complex relationships (e.g., nonlinear and non-monotonic relationships) between model predictions and individual parameters.

For most of the parameters, it is difficult to estimate the correlations among them based on available data, thus the correlation values are simply a best estimate from expert opinion. In this study, we assume a rank correlation of 0.3 between nominal survival rates for female and male adults, between nominal survival rates for larvae and pupae, between the survival factor under high sun exposure and that under high saturation deficit, between the short-range dispersal probabilities for females and males, and also between long-range dispersal probabilities for females and males. A correlation coefficient of 0.89 is assigned between intercept and slope for lipid prediction (see eq. (S2.6) in Text S2), which is based on the data fitting using a linear regression. All other parameter combinations are considered as being independent of each other because we lack information that clearly suggests that a correlation exists.

**References:**

1. Styer LM, Carey JR, Wang JL, Scott TW (2007) Mosquitoes do senesce: Departure from the paradigm of constant mortality. Am J Trop Med Hyg 76: 111-117.

2. Maciel-De-Freitas R, Codego CT, Lourenco-De-Oliveira R (2007) Body size-associated survival and dispersal rates of *Aedes aegypti* in Rio de Janeiro. Med Vet Entomol 21: 284-292.

3. Harrington LC, Edman JD, Scott TW (2001) Why do female *Aedes aegypti* (Diptera: Culicidae) feed preferentially and frequently on human blood? J Med Entomol 38: 411-422.

4. Harrington LC, Vermeylen F, Jones JJ, Kitthawee S, Sithiprasasna R, et al. (2008) Age-dependent survival of the dengue vector *Aedes aegypti* (Diptera : Culicidae) demonstrated by simultaneous release-recapture of different age cohorts. J Med Entomol 45: 307-313.

5. McDonald PT (1977) Population characteristics of domestic *Aedes aegypti* (Diptera: Culicidae) in villages on Kenya coast. 1. Adult survival and population size. J Med Entomol 14: 42-48.

6. Sheppard PM, Macdonal.Ww, Tonn RJ, Grab B (1969) Dynamics of an adult population of *Aedes aegypti* in relation to dengue haemorrhagic fever in Bangkok. J Anim Ecol 38: 661-702.

7. Gilpin ME, McClelland GAH (1979) Systems-analysis of the yellow fever mosquito *Aedes aegypti*. Forts Zool 25: 355-388.

8. Sharpe PJH, DeMichele DW (1977) Reaction kinetics of poikilotherm development. J Theor Biol 64: 649-670.

9. Tun-Lin W, Burkot TR, Kay BH (2000) Effects of temperature and larval diet on development rates and survival of the dengue vector *Aedes aegypti* in north Queensland, Australia. Med Vet Entomol 14: 31-37.

10. Rueda LM, Patel KJ, Axtell RC, Stinner RE (1990) Temperature-dependent development and survival rates of *Culex quinquefasciatus*  and *Aedes aegypti* (Diptera: Culicidae). J Med Entomol 27: 892-898.

11. Focks DA, Haile DG, Daniels E, Mount GA (1993) Dynamic life table model of *Aedes aegypti* (Diptera: Culicidae) - Analysis of the literature and model development. J Med Entomol 30: 1003-1017.

12. Farnesi LC, Martins AJ, Valle D, Rezende GL (2009) Embryonic development of *Aedes aegypti* (Diptera: Culicidae): influence of different constant temperatures. Mem Inst Oswaldo Cruz 104: 124-126.

13. Otero M, Schweigmann N, Solari HG (2008) A stochastic spatial dynamical model for *Aedes aegypti*. Bull Math Biol 70: 1297-1325.

14. Kamimura K, Matsuse IT, Takahashi H, Komuka J, Fukuda T, et al. (2002) Effect of temperature on the development of *Aedes aegypti* and *Aedes albopictus*. Med Entomol Zool 53: 53-58.

15. Silver JB (2008) Mosquito Ecology. New York, USA: Springer. 1477 p.

16. Harrington LC, Scott TW, Lerdthusnee K, Coleman RC, Costero A, et al. (2005) Dispersal of the dengue vector *Aedes aegypti* within and between rural communities. Am J Trop Med Hyg 72: 209-220.

17. Magori K, Legros M, Puente ME, Focks DA, Scott TW, et al. (2009) Skeeter Buster: a stochastic, spatially-explicit modeling tool for studying *Aedes aegypti* population replacement and population suppression strategies. Plos Neglect Trop Dis 3: e508.
