# Supplementary material for: Understanding Uncertainties in Model-Based Predictions of Aedes aegypti Population Dynamics
Source: PLoS Negl Trop Dis. 2010 Sep 28;4(9):e830. doi: 10.1371/journal.pntd.0000830 (PMC2946899; doi:10.1371/journal.pntd.0000830)
Supplement: Text S2 — Parameter estimation for the larval weight gain model. (0.09 MB DOC) [file pntd.0000830.s002.doc]

# Text S2: Parameter estimation for the larval weight gain model

The larval weight gain model [1] is governed by two equations:

$\begin{matrix} \frac{dW(t)}{dt}=aW(t)^{b}(1-e^{-cF(t)}) \\ \frac{dF(t)}{dt}=-\frac{1}{a}N(t)\frac{dW(t)}{dt} \end{matrix}$ (S2.)

where$N(t)$is the total number of larvae in the container, $W(t)$is the larval weight (mg), andis the amount of food (mg) in the container at time *t*. The factor$a$is the conversion rate of consumed food to biomass, *b* represents the body weight effect on larval food exploitation rate, and *c* is the coefficient of food dependence, with a lower value indicating a stronger effect of food on larval growth (see Figure S2.1 for more explanations) and a stronger effect of density dependence on larval population growth.

Larval growth rate relative to no food limitation

C=0.2

C=0.1

Food in container (mg)

C=0.5

Figure S2.1 Effect of the food amount in water containers on larval growth with different coefficients of food dependence based on the larval weigh gain model as specified in eq.(S2.1). A lower value of the coefficient of food dependence leads to a stronger effect of food on larval growth.

In order to estimate the three model parameters in eq. (S2.1), we assume that the observed data can be described by log-normal distributions as follows

$\log w(t)\sim N(\log w(t)_{p},\sigma^{2})$. (S2. )

In other words, the logged larval weight is normally distributed with mean [i.e.,$\log w(t)_{p}$] predicted by the larval weight gain model and variance $\sigma^{2}$. The experiment use three different levels food inputs (0.25 g, 0.1 g and 0.04 g liver powder in a 200 ml cup), four levels of larvae inputs (8, 20, 51 and 128), two strains (house and bush strain). With weight sampling at 12 hour intervals, there are 160 weight observation data points. Observations made at the first time point for each experiment are taken as initial values for the weight gain model in eq. (S2.1).

We employ a Bayesian approach to estimate model parameters. The prior distributions for the parameters are specified as follows,

$\begin{matrix} a\sim\text{Uniform}[0,0.5] \\ b\sim\text{Uniform}[0,1] \\ c\sim\text{Uniform}[0,1] \\ \frac{1}{\sigma^{2}}\sim\text{Gamma}(0.001,0.001) \end{matrix}$ (S2.)

where Uniform[$\alpha$,$\beta$] represents a uniform distribution with on the interval [$\alpha$,$\beta$]; Gamma ($\alpha$,$\beta$) represents a gamma distribution. With the specified prior distribution for parameters and the statistical model for data specified in eq.(S2.2), Bayes’ rule is used to derive the posterior distribution for the parameters given the observed data as follows,

$\begin{matrix} f(a,b,c,\sigma^{2}|w_{1},....,w_{n}) \\ \propto f(w_{1},....,w_{n}|a,b,c,\sigma^{2})f(a)f(b)f(c)f(\sigma^{2}) \\ =(\frac{1}{\sqrt{2\pi\sigma^{2}}})^{n}\exp[-\frac{1}{2\sigma^{2}}\sum_{i=1}^{n} (\log w_{i}-\log w_{ip})^{2}]f(a)f(b)f(c)f(\sigma^{2}) \end{matrix}$ (S2.)

where $w_{1},....,w_{n}$ are the observed body weights and $w_{ip}$are the predicted larval weights. The function $f(a),f(b),f(c),\text{and} f(\sigma^{2})$ are the prior probability distribution functions defined in eq. (S2.3). Since the posterior distribution in eq. (S2.4) can be very difficult to derive analytically, in this study, the Metropolis-Hastings approach [2,3] is used to draw samples for the parameters from the posterior distribution. Specifically, the algorithm is implemented as follows,

1. Assign initial values to $a,b,c,\sigma^{2}$;
2. Run the weight gain model in eq. (S2.1), which is numerically evaluated using the Euler method;
3. Calculate the posterior likelihood $f(a,b,c,\sigma^{2}|w_{1},....,w_{n})$ based on eq. (S2.4);
4. Propose new values for parameters with a multivariate normal distribution $a=a*,b=b*,c=c*,\sigma^{2}=\sigma^{2}*$;
5. Calculate the posterior likelihood $f(a*,b*,c*,\sigma^{2}*|w_{1},....,w_{n})$ based on eq. (S2.4);
6. Draw a random sample *u* from uniform [0,1]. If

$u<\frac{f(a*,b*,c*,\sigma^{2}*|w_{1},....,w_{n})}{f(a,b,c,\sigma^{2}|w_{1},....,w_{n})}$,

then accept the new proposed parameter values; otherwise stay put.

1. Repeat step 4)-6).

It can be shown that sample drawn by the Metropolis-Hastings method will follow the posterior distribution using the fact that the Markov chain is stationary if the proposal distribution is symmetric [4].

In this study, we run a chain of 150,000 iterations and a burn size of 50, 000 (the initial sequence of samples that is discarded to eliminate dependence on the initial choice of parameter values). We calculate the statistics of estimated parameters using every tenth sample of the parameters (to reduce the effect of auto-correlation on sample statistics) (See Table S2.1 ).

Table S2.1 Estimated parameters for the weight gain model

|  | *a* | *b* | *c* | $\sigma^{2}$ |
| --- | --- | --- | --- | --- |
| mean | 0.32 | 0.80 | 0.55 | 20.07 |
| Standard deviation | 0.0077 | 0.0060 | 0.25 | 2.25 |

The weight gain model in eq.(S2.1) specifies the net growth of larval weight. A full version of the weight gain model has an additional term for metabolic weight loss as follows,

$\frac{dW(t)}{dt}=a [W(t)^{b}(1-e^{-cF(t)})-d_{1}W(t)^{d_{2}}]$ (S2.)

where represents biomass loss due to metabolic activity. *d*_1_ is the coefficient of metabolic weight loss, with a higher value indicating higher amount of energy is used for metabolic activity, and *d*_2_ represents the effect of body weight on metabolic activity and is commonly set at 2/3 [5]. Since the metabolic weight loss term in the larval weight gain model (i.e., $d_{1}W(t)^{d_{2}}$) is not identifiable based on the available data, the coefficient of metabolic weight loss (i.e., *d*_1_) was assigned with a range between 0.005 and 0.032 based on expert opinion. This means that the percent of weight loss by metabolic activities is between 0.5 and 3.2 percent of body weight gain with no food constraint.

If there is no food available in the container, then the amount of lipid reserve in larva’s body can be crucial for survival. In Skeeter Buster, the amount of lipid is calculated using the following linear function of the logged larval weight, obtained by applying linear regression to data from Gilpin and McClelland [5],

(S2.)

where *L*(*t*) is the percentage of lipid weight in the larva’s body at time *t*. The and are the slope and intercept, respectively. The mean and standard deviations of and (see Table S4) are estimated based on fitting of the linear regression to the data from Gilpin and McClelland [5].

**References:**

1. Gilpin ME, McClelland GAH (1979) Systems-analysis of the yellow fever mosquito *Aedes aegypti*. Forts Zool 25: 355-388.

2. Metropolis N, Rosenbluth AW, Rosenbluth MN, Teller AH, Teller E (1953) Equation of state calculations by fast computing machines. J Chem Phys 21: 1087-1092.

3. Hastings WK (1970) Monte Carlo sampling methods using Markov chains and their applications. Biometrika 57: 97-109.

4. Gelman A (2004) Bayesian data analysis. Boca Raton, Fla.: Chapman & Hall/CRC. xxv, 668 p. p.

5. Gilpin ME, McClelland GAH (1979) Systems-analysis of the yellow fever mosquito *Aedes aegypti*. Forts Zool 25: 355-388.
