# Supplementary material for: Understanding Uncertainties in Model-Based Predictions of Aedes aegypti Population Dynamics
Source: PLoS Negl Trop Dis. 2010 Sep 28;4(9):e830. doi: 10.1371/journal.pntd.0000830 (PMC2946899; doi:10.1371/journal.pntd.0000830)
Supplement: Text S3 — Parameter estimation for the enzyme kinetics model. (0.07 MB DOC) [file pntd.0000830.s003.doc]

# Text S3: Parameter estimation for the enzyme kinetics model

The developmental rates of different life stages in the Skeeter Buster model are simulated based on an existing enzyme kinetics model [1]. This enzyme kinetics model assumes that development rate is determined by a single rate-controlling enzyme and that the enzyme is denatured at high and low temperatures. A simplified version of this model [2] is used in the Skeeter Buster model, assuming inactivation only at high temperatures. The developmental rate is calculated based on a nonlinear equation with four parameters,

$r(T_{t})=\frac{\rho_{(25^{^{\circ}}\text{C})}\frac{T_{t}}{298}e^{\frac{\Delta H_{\text{A}}^{\neq}}{R}(\frac{1}{298}-\frac{1}{T_{t}})}}{1+e^{\frac{\Delta H_{\text{H}}}{R}(\frac{1}{T_{1/2\text{H}}}-\frac{1}{T_{t}})}}$, (S3.1)

where *r*(*T_t_*) is the developmental rate (hr^-1^) at temperature *T* (K) on day *t*, *T_t_* is the water temperature for all immature stages and air temperature for adults. ρ(25ºC) is the development rate (hr^-1^) at 25ºC assuming no temperature inactivation of the critical enzyme; Δ*H*_A_^≠^ is the enthalpy of activation of the reaction catalyzed by the enzyme (cal/mol); Δ*H*_H_ is the enthalpy change associated with high temperature inactivation of the enzyme (cal/mol); and *T*_1/2H_ (K) is the temperature at which 50% of the enzyme is inactivated from high temperature.

In order to estimate the model parameters (*T_t_* , ρ(25ºC) , *T*_1/2H_ and Δ*H*_A_^≠^), we assume the observed data follow normal distributions as follows

$D(T)\sim N(D(T)_{p},\sigma^{2}(T))$ (S3.2)

where $D(T)_{p}$is the predicted development time at temperature *T* using eq. (S3.1) and $\sigma^{2}(T)$ is the standard deviation of the observed development times at temperature *T* . We use development time data at different temperatures from Farnesi et al.[3] to estimate the embryonic development rate (see Figure S1.3 in Text S1). Since the standard deviation for mean development time is very low due to the well-controlled laboratory conditions of the experiment, we increase the standard error proportionally based on data utilized by Focks et al. [4] to estimate egg development time to reflect the view that the field environment can cause higher variability in development times. The development times for larvae and pupae are estimated based on data from Rueda et al [5] and Tun-Lin et al [6]. The means and standard errors at different temperatures are weighted for different data sources with the weights chosen to be proportional to the sample sizes. The development times of gonotrophic cycles are estimated using data from Focks et al [4].

We employ a Bayesian approach to estimate model parameters. The prior distributions for parameters are specified as follows,

$\begin{matrix} T_{t\text{ }}\sim\text{Uniform }[300,330] \\ \rho_{\left(25^{\text{o}}\text{C}\right)}\sim\text{Uniform }[0,1] \\ \Delta H_{\text{A}}^{\neq}\sim\text{Uniform }[1000,200000] \\ \Delta H_{\text{H}}\sim\text{Uniform }[1000,200000]. \end{matrix}$ (S3.3)

Based on the specified prior distribution for model parameters and the statistical model for data in eq. (S3.2), Bayes’ rule is used to derive the posterior distribution for the parameters given the data as follows,

(S3.4)

where $D_{1},....,D_{n}$ and $\sigma_{1},....,\sigma_{n}$are the mean and standard deviations of observed development times at different temperatures.$f(T_{t\text{ }}),f(\rho_{\left(25^{\text{o}}\text{C}\right)}),f(\Delta H_{\text{A}}^{\neq}), \text{and} f(\Delta H_{\text{H}})$ are the prior distribution function as defined in eq. (S3.3). Similar to the estimation of growth model parameters, the Metropolis-Hastings approach is used to draw samples for parameters in the enzyme kinetics model from the posterior distribution in eq. (S3.4). In this study, we run a chain of 150,000 iterations and a burn size of 50, 000. The estimated statistics for the posterior distribution (sampled every 50 steps on the Markov chain sequence) are shown in Table 3.1.

Table S3.1 Estimated parameters for development times of different life stages obtained using the Bayesian approach described in the text (Values in parentheses are standard errors).

| *Life Stages* | *Parameters* | | | |
| --- | --- | --- | --- | --- |
|  | ρ(25ºC) | Δ*H*_A_^≠^ | *T*_1/2H_ | Δ*H*_H_ |
| Eggs | 0.34  (0.06) | 14265  (5518) | 312.17  (6.73) | 88762  (55073) |
| Larvae | 0.201  (0.049) | 26372  (9540) | 305.61  (6.71) | 55648  (18910) |
| Pupae | 0.483  (0.121) | 15497  (3427) | 316.91  (7.90) | 40605  (29622) |
| Gonotrophic cycles | 0.233  (0.044) | 15106  (3513) | 319  (6.87) | 100375  (59600) |

Since we are more concerned with the overall contributions to variances of model outputs by the uncertainties in development rates as a functional curve (or a profile) of temperature rather than the contribution by individual parameters**,** we used a profile-based sampling approach to sample the development rate profiles. The main idea of the profile-based sampling is that, we use the mean development time under the temperature range between 24-28^o^C, which is the most relevant ranges in our study area, to draw random samples of development-rate profiles. Each development-rate profile will correspond to a mean development time (Figure S3.1). For the uncertainty analysis, instead of drawing samples for the parameters, we draw a sample for the mean development time between 24-28^o^C. For each mean development time drawn, we get corresponding parameter values for the enzyme kinetics model using a pool of 2000 development rate profiles (each with a specific set of parameter values) generated by the Metropolis-Hastings approach.

Temperature

Development time

Profile 1: Tt , ρ_(25ºC)_, ΔH_A≠_, and ΔH_H_

Mean development time 1

Temperature range

Profile 2: Tt’ , ρ_(25ºC)_’, ΔH’_A≠_, and ΔH’_H_

Mean development time 2

Figure S3.1 Illustration of mean development time within a specified temperature range as a surrogate for sampling of development time profiles. Development time profiles are profiles (or curves) assigning development rates under different temperatures, determined by an enzyme kinetics model in eq.(S3.1). Profile 1 and 2, both with a unique set of parameters (T_t_ , ρ_(25ºC)_, ΔH_A≠_, and ΔH_H_ ), are represented by the mean development time within the specified temperature range (24-28^o^C in this study). Each profile in a 2000 profile pool generated by a Metropolis-Hastings algorithm corresponds to a mean development time within the specified temperature range. For each sampled value of mean development time, a corresponding development profile in the pool with the same (or closest) mean development time will be selected to predict the development rate at different temperatures.

**References:**

1. Sharpe PJH, DeMichele DW (1977) Reaction kinetics of poikilotherm development. J Theor Biol 64: 649-670.

2. Schoolfield RM, Sharpe PJH, Magnuson CE (1981) Non-linear regression of biological temperature-dependent rate models based on absolute reaction-rate theory. J Theor Biol 88: 719-731.

3. Farnesi LC, Martins AJ, Valle D, Rezende GL (2009) Embryonic development of *Aedes aegypti* (Diptera: Culicidae): influence of different constant temperatures. Mem Inst Oswaldo Cruz 104: 124-126.

4. Focks DA, Haile DG, Daniels E, Mount GA (1993) Dynamic life table model of *Aedes aegypti* (Diptera: Culicidae) - Analysis of the literature and model development. J Med Entomol 30: 1003-1017.

5. Rueda LM, Patel KJ, Axtell RC, Stinner RE (1990) Temperature-dependent development and survival rates of *Culex quinquefasciatus*  and *Aedes aegypti* (Diptera: Culicidae). J Med Entomol 27: 892-898.

6. Tun-Lin W, Burkot TR, Kay BH (2000) Effects of temperature and larval diet on development rates and survival of the dengue vector *Aedes aegypti* in north Queensland, Australia. Med Vet Entomol 14: 31-37.
