# Supplementary material for: Understanding Uncertainties in Model-Based Predictions of Aedes aegypti Population Dynamics
Source: PLoS Negl Trop Dis. 2010 Sep 28;4(9):e830. doi: 10.1371/journal.pntd.0000830 (PMC2946899; doi:10.1371/journal.pntd.0000830)
Supplement: Text S4 — Quantification of stochastic uncertainty. (0.04 MB DOC) [file pntd.0000830.s004.doc]

# Text S4: Quantification of stochastic uncertainty

The predicted population density (for either a specific house or at the community level) for a given life stage *i* at time *t*, denoted *N_i_*(*t*), depends both on the parameter values of the model and on random noise (arising from demographic and environmental stochasticity). We can write

$$\text{N}_{i}\left(t\right)= u_{i}\left(t\right)+e_{i}\left(t\right)$$

where $u_{i}\left(t\right)$is the mean population size and $e_{i}\left(t\right)$ is the random noise. If we have two realizations of the model carried out using the same parameter values, then

$\text{N}^{\text{'}}_{i}\left(t\right)-\text{N}_{i}\left(t\right)= e_{i}\left(t\right)-e_{i}'\left(t\right).$

Calculating the variance of both sides of the above equation, we can show that

$Var[\text{e}_{i}\left(t\right)]=\text{Var[N}^{\text{'}}_{i}\left(t\right)-\text{N}_{i}\left(t\right)]/2$**.**

This indicates that we can estimate stochastic uncertainty by running the model twice for each parameter set sampled by FAST. Namely,

$V\hat{a}r[\text{e}_{i}\left(t\right)]=\frac{1}{2n}\sum_{i=1}^{n} \text{[D}_{\text{N}_{i}}^{\text{(j)}}\left(t\right)-\bar{\text{D}}_{\text{N}_{i}}^{\text{(j)}}\left(t\right)]^{2}$

where $\text{D}_{\text{N}_{i}}^{\text{(j)}}\left(t\right)$ indicates the difference between two replicates for the *j*th FAST sample [$\text{D}_{\text{N}_{i}}^{\text{(j)}}\left(t\right)=\text{N}^{\text{'}}_{i}\left(t\right)-\text{N}_{i}\left(t\right)$]; and *n* is the FAST sample size (5000 in our study). Finally, we can use the ratio of $V\hat{a}r[\text{e}_{i}\left(t\right)]$ to the total variance of $\text{N}_{i}\left(t\right)$ to measure the proportion of stochastic uncertainty in the population density prediction at the community or individual-house level.
