# Supplementary material for: Understanding Uncertainties in Model-Based Predictions of Aedes aegypti Population Dynamics
Source: PLoS Negl Trop Dis. 2010 Sep 28;4(9):e830. doi: 10.1371/journal.pntd.0000830 (PMC2946899; doi:10.1371/journal.pntd.0000830)
Supplement: Text S5 — Spatial statistics. (0.04 MB DOC) [file pntd.0000830.s005.doc]

# Text S5: Spatial statistics

In this study, we use three spatial statistics (Moran’s *I*, Getis G*_i_** (*d*) and semivariogram) to measure population distribution pattern. The Moran’s Index, *I,* was proposed by Moran in 1950 to evaluate whether a spatial pattern is clustered, dispersed, or random [1]. A Moran's Index value near +1.0 indicates clustering, an index value near -1.0 indicates dispersion, and an index of 0 indicates complete randomness. The specific formula for calculating the Moran’s *I* is as follows,

$$I=\frac{N}{\sum_{i} \sum_{j} w_{i,j}}\frac{\sum_{i} \sum_{j} w_{i,j}(x_{i}-\bar{x})(x_{j}-\bar{x})}{\sum_{i} (x_{i}-\bar{x})^{2}}$$

where *N* equals the number of observations; *w_ij_* is the weight between locations *i* and *j*; *x_i_* and *x_j_* are the values at locations *i* and *j*; $\bar{x}$ is the average over all locations of the variable. In this study, the weight *w_ij_* is proportion to the inverse distance between houses.

The Getis *G_i_**(*d*) statistic is used in this study to identify hot spots for food inputs at individual houses. The formula for *G_i_**(*d*) is as follows [2,3],

$$G_{i}^{*}(d)=\frac{\sum_{j=1}^{N} w_{ij}(d)x_{j}-\bar{x}\sum_{j=1}^{N} w_{ij}(d)x_{j}}{S\sqrt{[N\sum_{j=1}^{N} w_{ij}^{2}(d)-(\sum_{j=1}^{N} w_{ij}(d))^{2}]/(N-1)}}$$

where *w_ij_*(*d*) is the weight between locations *i* and *j* with a specified threshold distance *d,* which is used to specify the neighborhood size around of the house of interest to examine if this house is a local high/low density spot; and S is the standard deviation of all observations. In this study, we select *w_ij_*(*d*) based on the inverse distance throughout the study area (i.e., *d* is sufficiently big to incorporate all houses), which is same as that in the calculation of Moran's *I*. *G_i_**(*d*) has an asymptotic normal distribution. A *z*-score can be calculated to see if the population within a specific house is significantly higher/lower than its neighborhood.

The semivariogram is a function of distance describing the degree of spatial dependence of a spatial random process [4]. The formula is as follows,

$$r(h)=\frac{1}{\left|N(h)\right|}\sum_{i,j\in N(h)}^{N} \left|x_{i}-x_{j}\right|^{2}$$

where $N(h)$is the set of data point pairs ($x_{i}$, $x_{j}$) that are distance *h* apart and $\left|N(h)\right|$ represents the number of data point pairs. A higher value of $r(h)$indicates lower spatial autocorrelation. Generally, the spatial auto-correlation will decrease with distance *h* and finally stabilize. The range (i.e., the distance after which $r(h)$starts to stabilize) can be used to indicate the strength of spatial auto-correlation.

**References:**

1. Moran PAP (1950) Notes on continuous stochastic phenomena. Biometrika 37: 17-23.

2. Getis A, Ord JK (1992) The analysis of spatial association by use of distance statistics. Geogr Anal 24: 189-206.

3. Ord JK, Getis A (1995) Local spatial autocorrelation statistics - distributional issues and an application. Geogr Anal 27: 286-306.

4. Goovaerts P (1997) Geostatistics for natural resources evaluation. New York: Oxford University Press. 483 p.
