# Supplementary material for: Understanding Uncertainties in Model-Based Predictions of Aedes aegypti Population Dynamics
Source: PLoS Negl Trop Dis. 2010 Sep 28;4(9):e830. doi: 10.1371/journal.pntd.0000830 (PMC2946899; doi:10.1371/journal.pntd.0000830)
Supplement: Text S6 — Temporal variability of population density at the community level. (0.06 MB DOC) [file pntd.0000830.s006.doc]

# Text S6: Temporal variability of population density at the community level

In this section, we examine temporal variability in population density at the community level. Specifically, we assess contributions by different model parameters to uncertainty in the predicted temporal coefficient of variation (CV). The temporal coefficient of variation is calculated as follows: for each simulation run, we consider the community-level daily population densities seen over the second simulation year, calculating the standard deviation and mean of these densities. The CV is then calculated as the ratio of these two quantities. The temporal CV measures the relative temporal variability adjusted by population size. Temporal variability may result from stochastic uncertainty, biological development cycles, environmental factors (e.g., extreme temperature) and temporal dynamics of food. Since stochastic uncertainty is a major source of temporal variation, model parameters explain much less of uncertainties in temporal CV (generally less than 30% in total, see Figure S6.1 and S6.2) compared to population densities at the community level (see result section in the main text). For the egg density, the nominal survival rate for female adults and the gonotrophic development rate are two important parameters contributing to its temporal variability (Figure S6.1 a). The survival rate becomes an important parameter because it is a major factor affecting population size (see result section in the main text for details), which will affect the population’s susceptibility to stochasticity. Parameters important for temporal variability in predicted larval population density include the high temperature limit for nominal egg survival, the high temperature limit for predator activities, as well as the nominal survival rate for female adults (Figure S6.1 b). The high temperature limits are important for temporal variability in the predicted larval population density because the maximum water temperatures on some days can reach (see Figure S3 c) the high temperature limits for nominal egg survival (defined as being between 28^o^C to 35 ^o^C) and high temperature limits for predator activities (defined as being between 25^o^C to 35 ^o^C). Important parameters contributing to temporal variability in the predicted pupal population density include the survival rate for female adults, the low temperature limit for nominal survival of pupae/larvae, the pupal development rate, and the coefficient of food dependence (Figure S6.1 c). The low temperature limit for survival of larvae/pupae is important because the minimum water temperatures are partially covered by this low temperature limit with its uncertainty range defined between 10^o^C to 20 ^o^C (see Figure S3 b). The coefficient of food dependence is important because it will affect the food exploitation rate, which can affect the temporal dynamics of food in the containers and the strength of density-dependence effect on larval growth. Strong density dependence in larval growth as determined by a small value of the coefficient of food dependence will lead to more temporal variability in population dynamics (see Figure S6.3). However, when the coefficient of food dependence is larger than 0.4, it tends to have much a less effect on temporal variability in population dynamics. This is because density dependence in larval growth become weak and food will only have effects on body growth when the food in container is very low (see Figure S2.1 in Text S2 for a better understanding).

For nulliparous female adults, the low temperature limit for nominal survival of pupae and larvae, and the survival rate of female adults are important parameters contributing to its temporal variability (Figure S6.2 a). Because the population dynamics of parous female adults is mainly affected by their survival rate (see result section in main text), the survival rate is also a dominant factor affecting the temporal variability (Figure S6.2 b), due to the fact that the population size can affect the population’s susceptibility to stochasticity (see result section in the main text for details). Other important parameters contributing to the temporal variability in predicted parous female adult density include the gonotrophic development rate, the nominal survival rate for male adults, and the low temperature limit for nominal survival of larvae/pupae. For temporal variability of male adults, important parameters include the nominal survival rates for female and male adults as well as the low temperature limit for nominal survival of larvae and pupae (Figure S6.2 c).

(a): Eggs [31.7 %]

(b): Larvae [38.2 %]

(c): Pupae [16.0 %]

x1: Nominal daily survival rate for female adults

x2: Low temperature limit for nominal survival of pupae and larvae

x3: Pupal development rate

x4: Coefficient of food dependence for larvae

x5: Nominal daily survival rate for larvae

x1: High temperature limit for nominal egg survival

x2: High temperature limit for predator activities

x3: Nominal daily survival rate for female adults

x4: Nominal daily survival rate for larvae

x5: Low temperature limit for nominal survival of pupae and larvae

x6: Predator-dependent egg survival under high temperature

x7: Low temperature limit for predator-dependent egg survival

x1: Nominal daily survival rate for female adults

x2: Gonotrophic development rate

x3: Physiological development threshold for later gonotrophic cycles

x4: Low temperature limit for nominal survival of pupae and larvae

x5: Nominal daily survival rate for larvae

x6: Minimum temperature for oviposition

x7: High temperature limit for predator-dependent egg survival

Figure S6.1 Uncertainty contributions by different model parameters for the temporal coefficient of variation (CV) of (a) eggs, (b) larvae and (c) pupae during the second simulation year. The vertical bars represent the 95% confidence intervals. The percentage values in brackets represent overall percentages of variance explained by the parameters shown in the figure.

(a): Nulliparous female adults [18.4 %]

(b): Parous female adults [53.9 %]

(c): Male adults [24.9 %]

x1: Nominal daily survival rate for female adults

x2: Low temperature limit for nominal survival of pupae and larvae

x3: Nominal daily survival rate for male adults

x4: Pupal development rate

x5: Nominal daily survival rate for larvae

x6: Embryonic development rate

x7: Larval development rate

x1: Nominal daily survival rate for female adults

x2: Gonotrophic development rate

x3: Nominal daily survival rate for male adults

x4: Low temperature limit for nominal survival of pupae and larvae

x5: Nominal daily survival rate for larvae

x6: Embryonic development rate

x1: Low temperature limit for nominal survival of pupae and larvae

x2: Nominal daily survival rate for female adults

x3: Gonotrophic development rate rate

x4: Nominal daily survival rate for larvae

Figure S6.2 Uncertainty contributions by different model parameters for the temporal coefficients of variations of (a) nulliparous female adults, (b) parous female adults and (c) male adults during the second simulation year. The vertical bars represent the 95% confidence intervals. The percentage values in brackets represent overall percentages of variance explained by the parameters shown in the figure.

Figure S6.3 Dependence of temporal variability in population density at the community level on the coefficient of food dependence. The coefficient of food dependence accounts for about 1.3 % of uncertainty in temporal variability as measured by the temporal coefficient of variation during the second simulation year. The curves are fitted to the scatter plot of parameter values sampled by FAST and the corresponding predicted coefficient of variation in pupal density during the second simulation year using cubic smoothing splines with the SemiPar R package [1]. The shaded areas are the 95% confidence intervals of the fitted lines.

**References**

1. Wand MP, Coull BA, French JL, Ganguli B, Kammann EE, et al. (2005) SemiPar 1.0. R package. http://cran.r-project.org.
