# Supplementary material for: Understanding Uncertainties in Model-Based Predictions of Aedes aegypti Population Dynamics
Source: PLoS Negl Trop Dis. 2010 Sep 28;4(9):e830. doi: 10.1371/journal.pntd.0000830 (PMC2946899; doi:10.1371/journal.pntd.0000830)
Supplement: Text S7 — Temporal variability of population density at the individual-house level. (0.63 MB DOC) [file pntd.0000830.s007.doc]

# Text S7: Temporal variability of population density at the individual-house level

For uncertainty in the temporal CV (calculated with a time interval of 15 days instead of the daily interval due to the large data storage requirement for densities of each life stage over 730 days for 5000 simulations) of population density at the individual-house level, our results show that CVs are relatively high where there is low population density (Figure S7.1 a and S7.2 a). The nominal survival rates of female adults and larvae and the coefficient of metabolic weight loss, which are important parameters contributing to population density, are also important parameters contributing to temporal variability (Figure S7.1 b, c , d and Figure S7.2 b, c , d). In contrast to the temporal variability in population density at the community level, our results show that spatial dispersal can be an important parameter contributing to the temporal variability at the individual-house level (Figure S7.2 f), with higher spatial dispersal probability leading to lower temporal variability in the population dynamics (see Figure S7.3). Spatial dispersal also contributes to the temporal variability in pupal population density, but to a much lesser extent (see Figure S7.1 f). The coefficient of food dependence is very important for the temporal variability of pupal population density in houses with relatively small food input and relatively high egg density (see Figure S7.2 g), where there are stronger density-dependence effects on larval growth. The low temperature limit (10-20^o^C) for nominal survival of pupae/larvae, which is important for temporal variability at the community level, is not detected as an important parameter at the individual-house level. This is due to the fact we use a longer time interval (15 days) to calculate the CVs, making occasional temporal variability (e.g., due to temperature falling below the low temperature limit for nominal survival of pupae and larvae) less detectable. Since the high temperature limit (25-35^o^C) for predator-dependent egg survival is more frequently exceeded for the weather in Iquitos, it is an important parameter contributing to temporal variability in population density at the individual-house level (Figure S7.2 e).

(a) Mean temporal CV based on 5000 FAST samples

(d) Proportion of uncertainty in temporal CV contributed by coefficient of metabolic activity

(g) Proportion of uncertainty in temporal CV contributed by coefficient of food dependence

(f) Proportion of uncertainty in temporal CV contributed by female adult short-range dispersal probability

(c) Proportion of uncertainty in temporal CV contributed by larval survival rate

(b) Proportion of uncertainty in temporal CV contributed by female adult survival rate

(e) Proportion of uncertainty in temporal CV contributed by high temperature limit for predator-dependent egg survival

Figure S7.1 Mean temporal coefficient of variation (CV) for pupal population density at the individual-house level (a) and the proportion of uncertainty in CV contributed by different model parameters (b-g). To simplify this figure, only parameters with maximum uncertainty contributions larger than 0.03 are plotted except for panel (f) for the comparison of the importance of mosquito dispersal for different life stages. We did not plot the proportion of uncertainty in CV contributed by the nominal survival rate for male adults since the uncertainty contribution is mainly due to its correlation with the nominal survival rate for female adults.

(a) Mean temporal CV based on 5000 FAST samples

(d) Proportion of uncertainty in temporal CV contributed by coefficient of metabolic activity

(e) Proportion of uncertainty in temporal CV contributed by high temperature limit for predator-dependent egg survival predation

(f) Proportion of uncertainty in temporal CV contributed by female adult short-range dispersal probability

(c) Proportion of uncertainty in temporal CV contributed by larval survival rate

(b) Proportion of uncertainty in temporal CV contributed by female adult survival rate

Figure S7.2 Mean temporal coefficient of variation (CV) for female adult population density at the individual-house level (a) and the proportion of uncertainty in CV contributed by different model parameters (b-f). To simplify this figure, only parameters with maximum uncertainty contributions larger than 0.03 are plotted. We did not plot the proportion of uncertainty in CV contributed by the nominal survival rate for male adults since the uncertainty contribution is mainly due to its correlation with the nominal survival rate for female adults.

Figure S7.3 Dependence of temporal variability in female adult population density at an specific individual house (located at 4^th^ row from top and 5^th^ column from left) on short-range spatial dispersal. The short-range dispersal accounts for 3.8% of uncertainty in the temporal variability as measured by the temporal coefficient of variation during the second simulation year. The curves are fitted to the scatter plot of parameter values sampled by FAST and the corresponding predicted coefficient of variation in pupal density during the second simulation year using a cubic smoothing splines with the SemiPar R package [1]. The shaded areas are the 95% confidence intervals of the fitted lines.

**References**

1. Wand MP, Coull BA, French JL, Ganguli B, Kammann EE, et al. (2005) SemiPar 1.0. R package. http://cran.r-project.org.
