# Supplementary figures and images for: Understanding Uncertainties in Model-Based Predictions of Aedes aegypti Population Dynamics
Source: PLoS Negl Trop Dis. 2010 Sep 28;4(9):e830. doi: 10.1371/journal.pntd.0000830 (PMC2946899; doi:10.1371/journal.pntd.0000830)

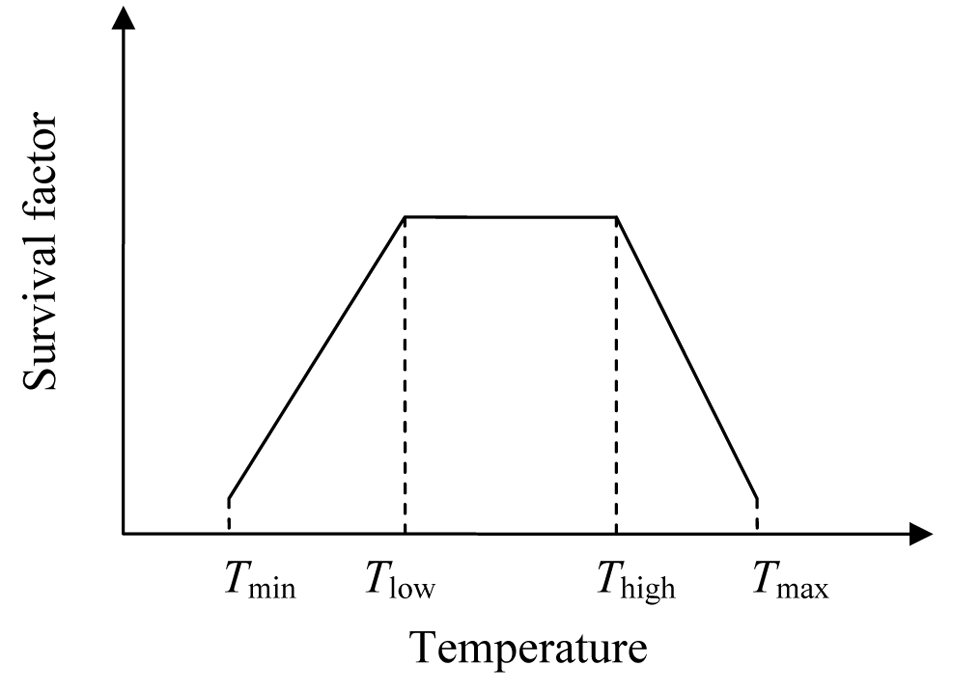

Supplement: Figure S1 — Survival factor as a function of temperature. The survival factor ranges between 0 and 1 and is multiplied with nominal survival rate to get the temperature-dependent survival rate. Tmin is the minimum temperature for survival, below which the low temperature has a strong effect on mosquito survival (the survival factor is generally less than 0.05); Tlow is the low temperature limit below which is suboptimal for mosquito survival; Thigh is the high temperature limit above which is suboptimal for mosquito surivival; Tmax is the maximum temperature for survival, above which the high temperature has a very strong effect on mosquito survival (the survival factor is generally less than 0.05). (0.07 MB TIF) [file pntd.0000830.s008.tif]

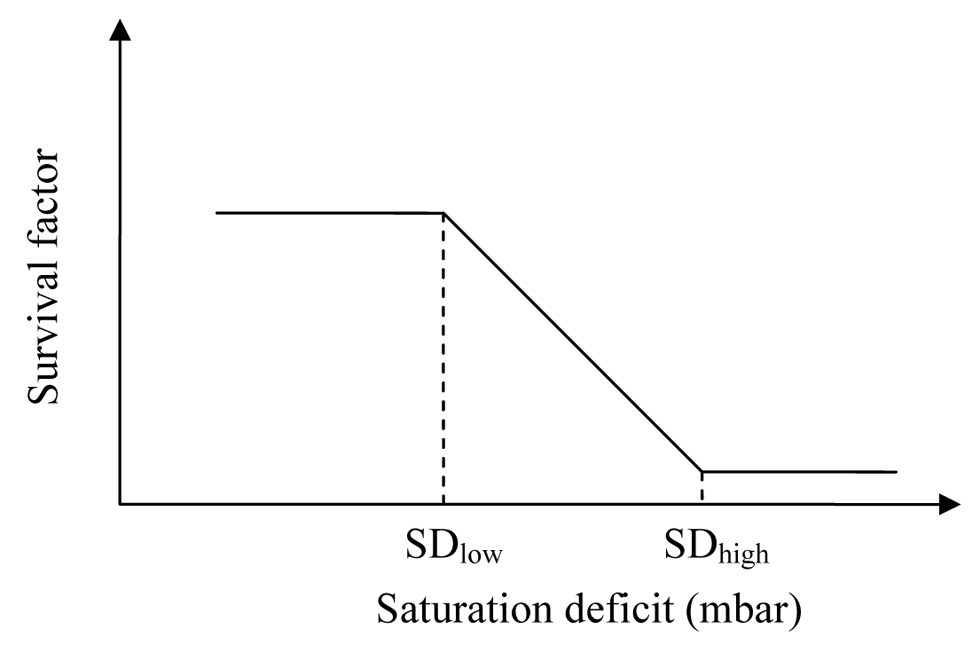

Supplement: Figure S2 — Survival factor as a function of saturation deficit (SD). The survival factor ranges between 0 and 1 and is multiplied with nominal survival rate to get the humidity-dependent survival rate. SDlow is the low saturation deficit limit below which saturation deficit has little effect on mosquito survival. The survival rate decreases linearly between SDlow and SDhigh, the high saturation deficit limit above which the saturation deficit has a strong effect on mosquito survival (survival factor is low). (0.06 MB TIF) [file pntd.0000830.s009.tif]

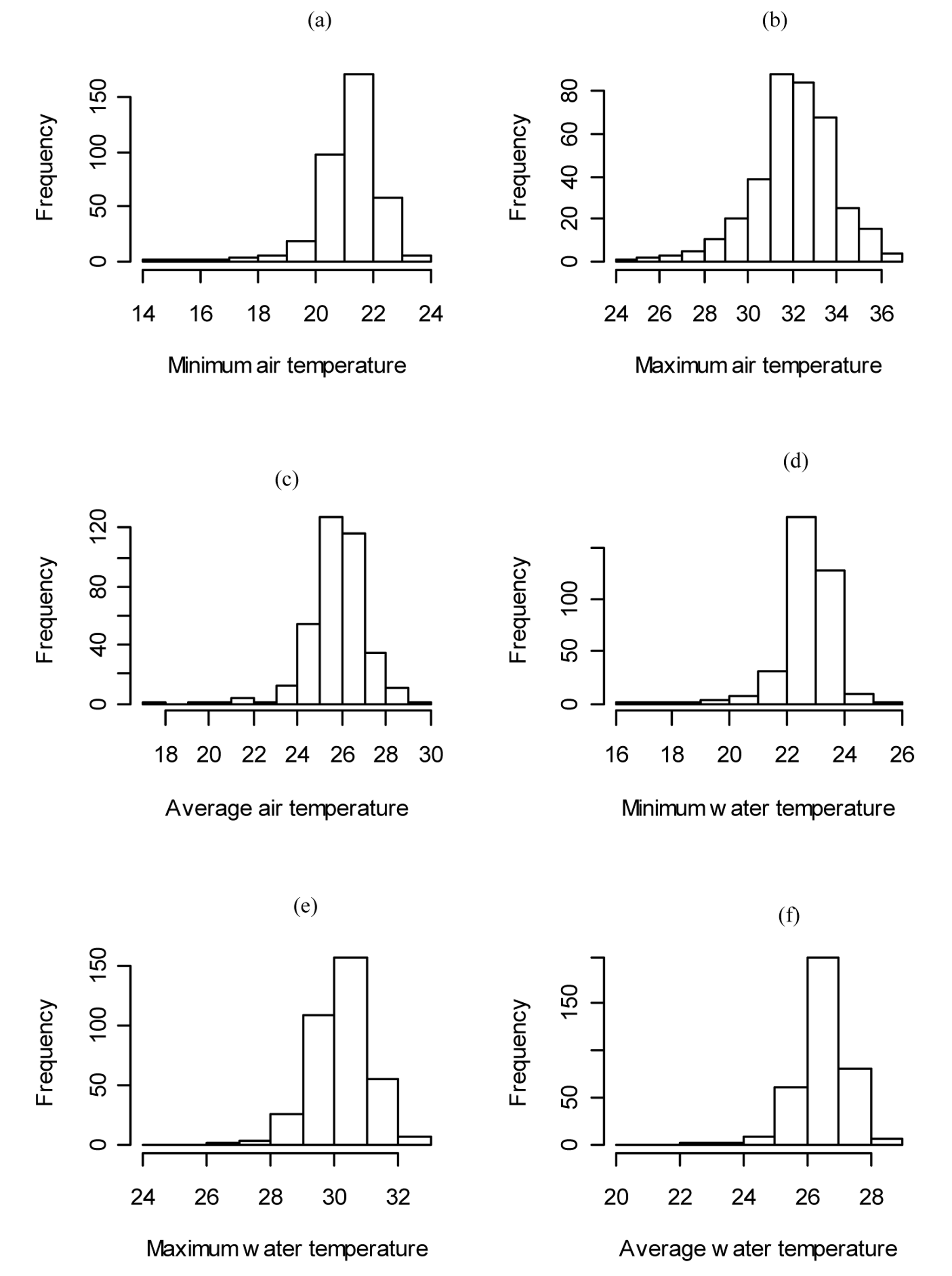

Supplement: Figure S3 — Histograms of air and water temperatures (degrees Celsius) in Iquitos for year 2000. The container water temperatures are simulated using a polynomial function obtained from a regression of water temperature on air temperature and sun exposure for 12 containers monitored for 76 days in Gainesville, FL, USA [4]. The water temperature is calculated assuming a sun exposure of 0.5 for the container. (0.49 MB TIF) [file pntd.0000830.s010.tif]

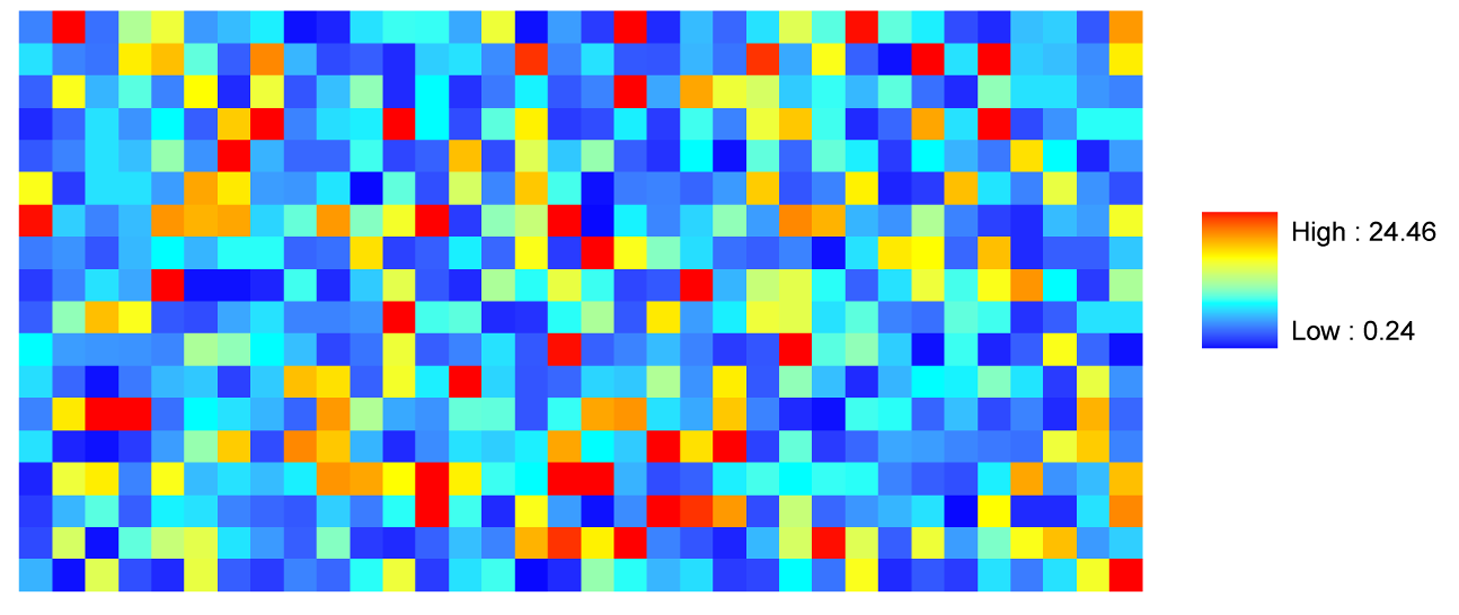

Supplement: Figure S4 — Sum of daily food input from different containers (Unit: mg/day) at individual houses. Each block/cell represents a single house. The food inputs are fitted to the pupal data in the mosquito survey at individual houses in Iquitos [23]. The food inputs are not spatially clustered based on the Moran's I statistic [46] using inverse distance weights (I = 0.005, p-value = 0.82). (0.21 MB TIF) [file pntd.0000830.s011.tif]

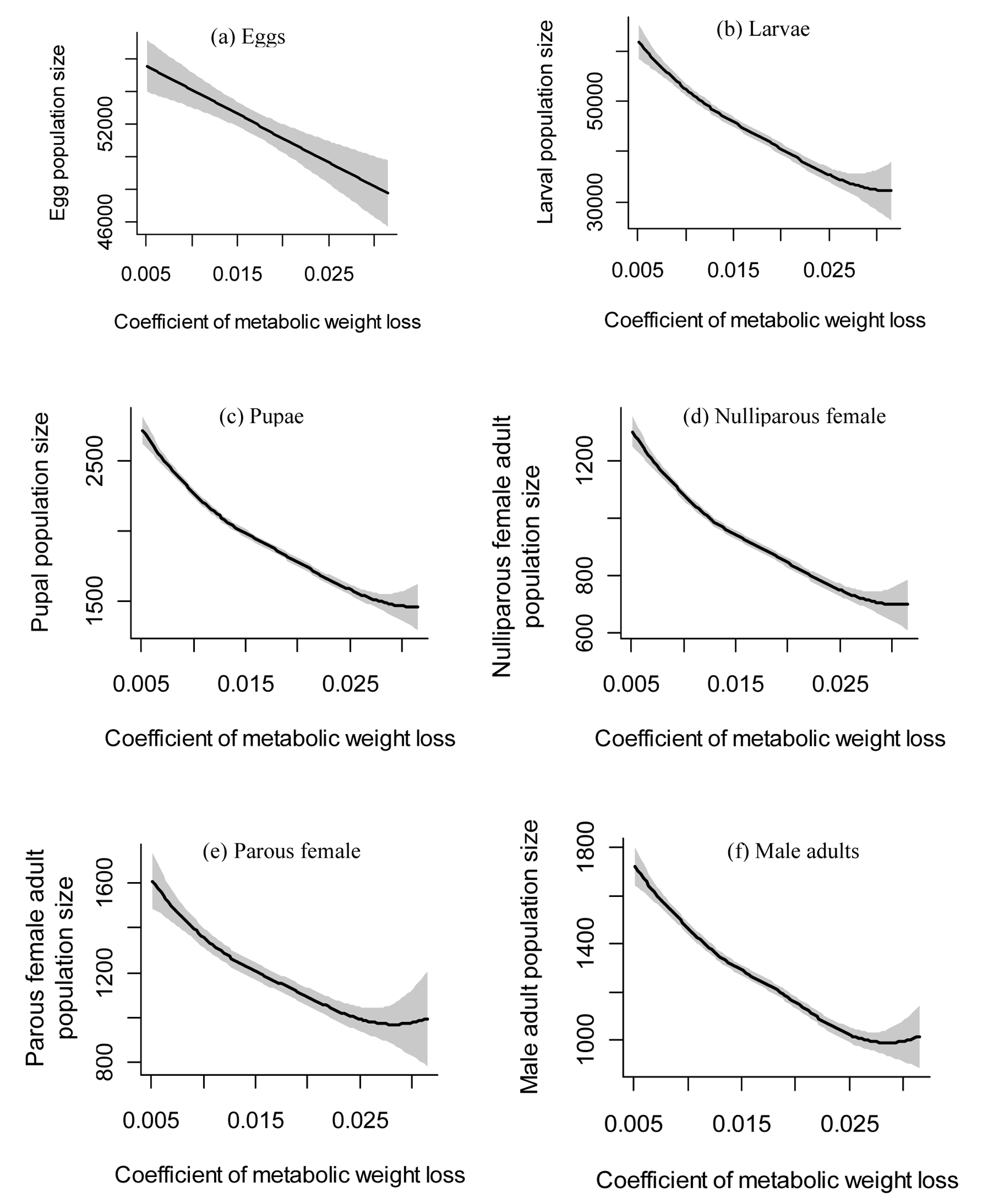

Supplement: Figure S5 — Dependence of community-level population density on coefficient of metabolic weight loss at different life stages. The curves are fitted to the scatter plot of parameter values sampled by FAST and the corresponding predicted population densities using cubic smoothing splines with the SemiPar R package [45]. The shaded areas are the 95% confidence intervals of the fitted lines. (0.58 MB TIF) [file pntd.0000830.s012.tif]

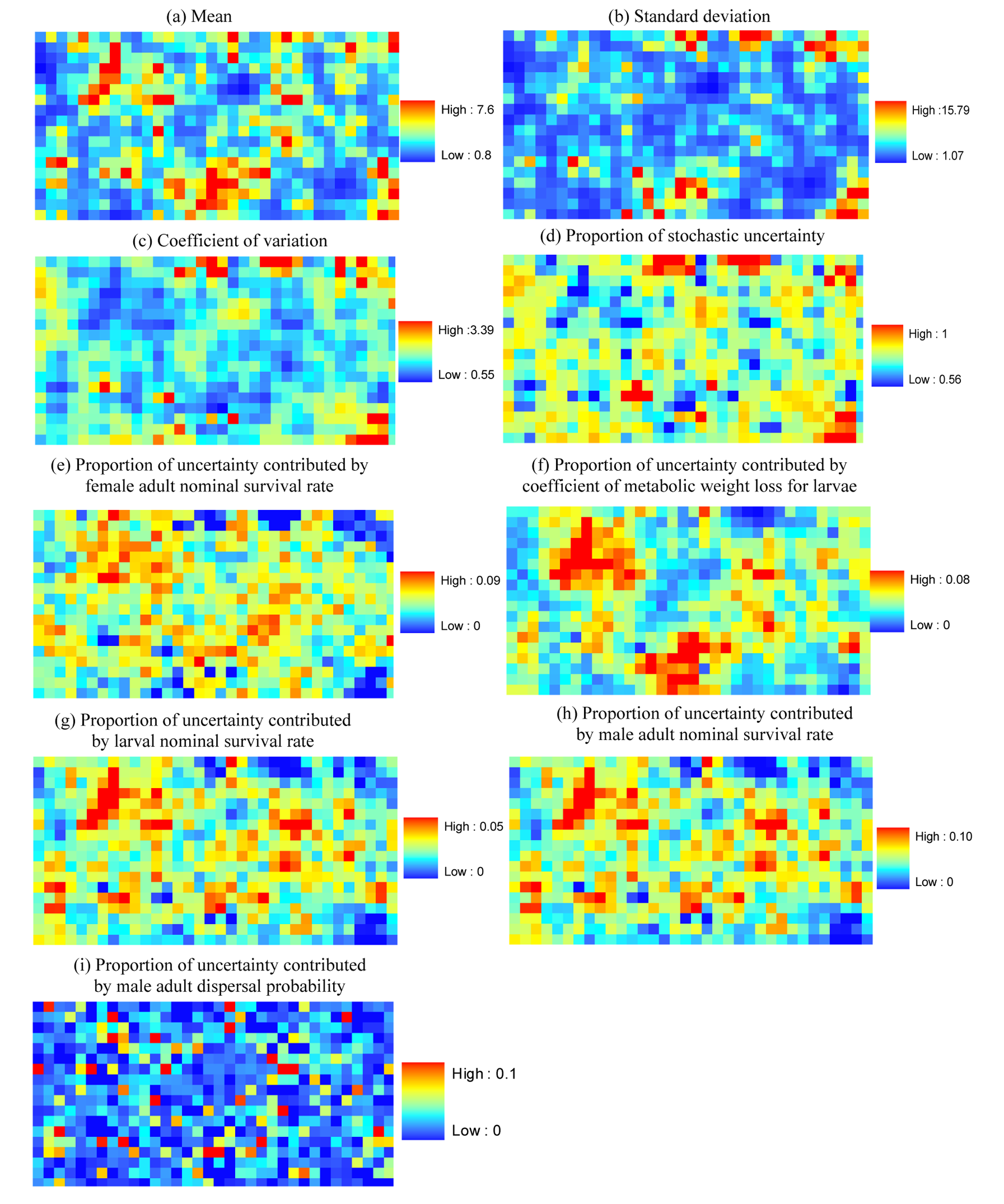

Supplement: Figure S6 — Uncertainty in the predicted male adult population density at the individual-house level on simulation day 720. For each individual house, we quantify uncertainty in the predicted population density (as is jointly described by the (a) mean, (b) standard deviation, and (c) coefficient of variation of predicted population density across the parameter sets sampled by FAST), (d) the proportion of uncertainty contributed by stochasticity, and (e–i) the proportions of uncertainty contributed by specific model parameters. To simplify this figure, only parameters with uncertainty contributions in any house larger than 5% are plotted. (1.47 MB TIF) [file pntd.0000830.s013.tif]

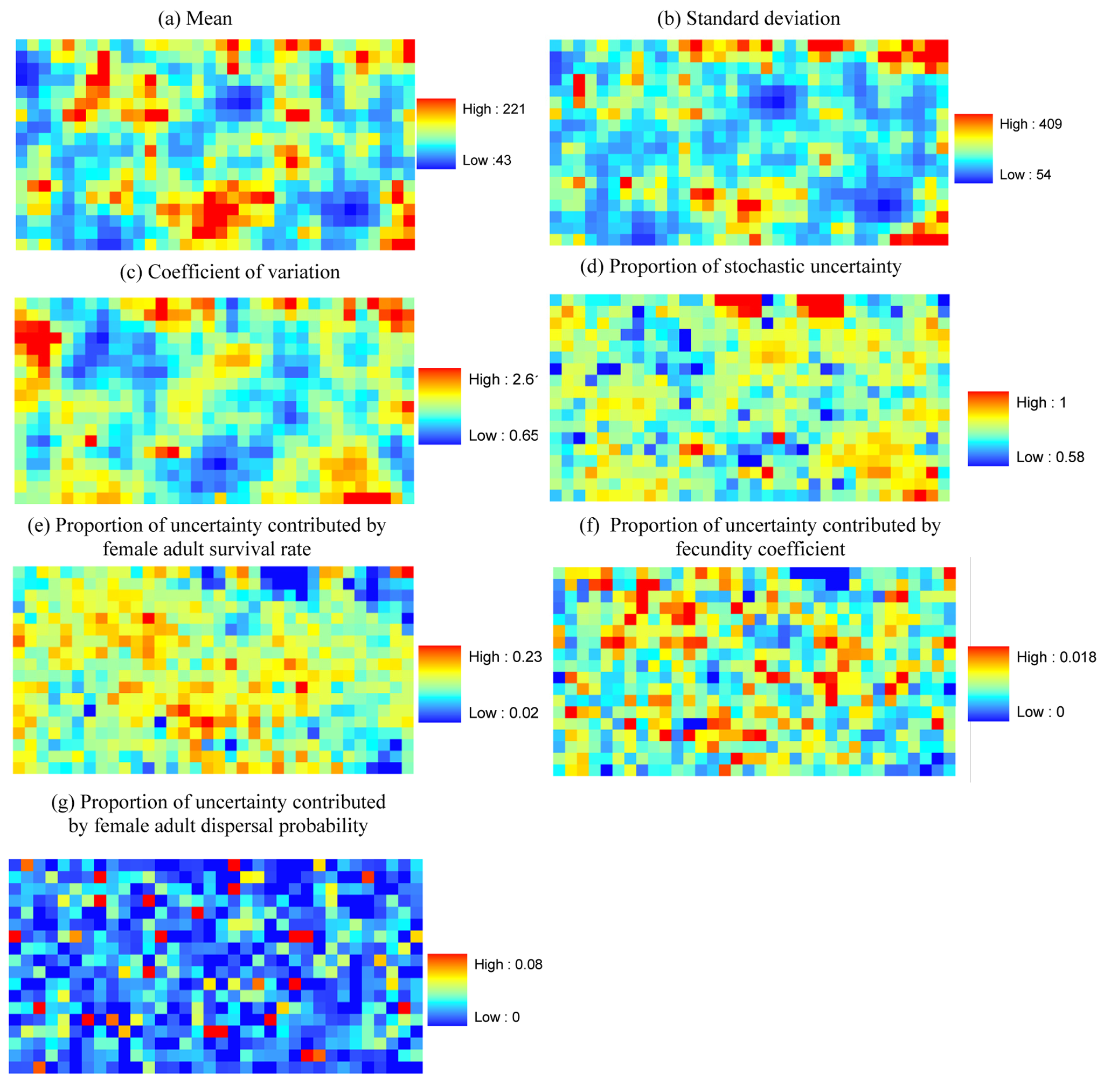

Supplement: Figure S7 — Uncertainty in the predicted egg density at the individual-house level on simulation day 720. For each individual house, we quantify uncertainty in the predicted population density (as is jointly described by the (a) mean, (b) standard deviation, and (c) coefficient of variation of predicted population density across the parameter sets sampled by FAST), (d) the proportion of uncertainty contributed by stochasticity, and (e–g) the proportions of uncertainty contributed by specific model parameters. To simplify this figure, only parameters with uncertainty contributions in any house larger than 5% are plotted. (1.75 MB TIF) [file pntd.0000830.s014.tif]

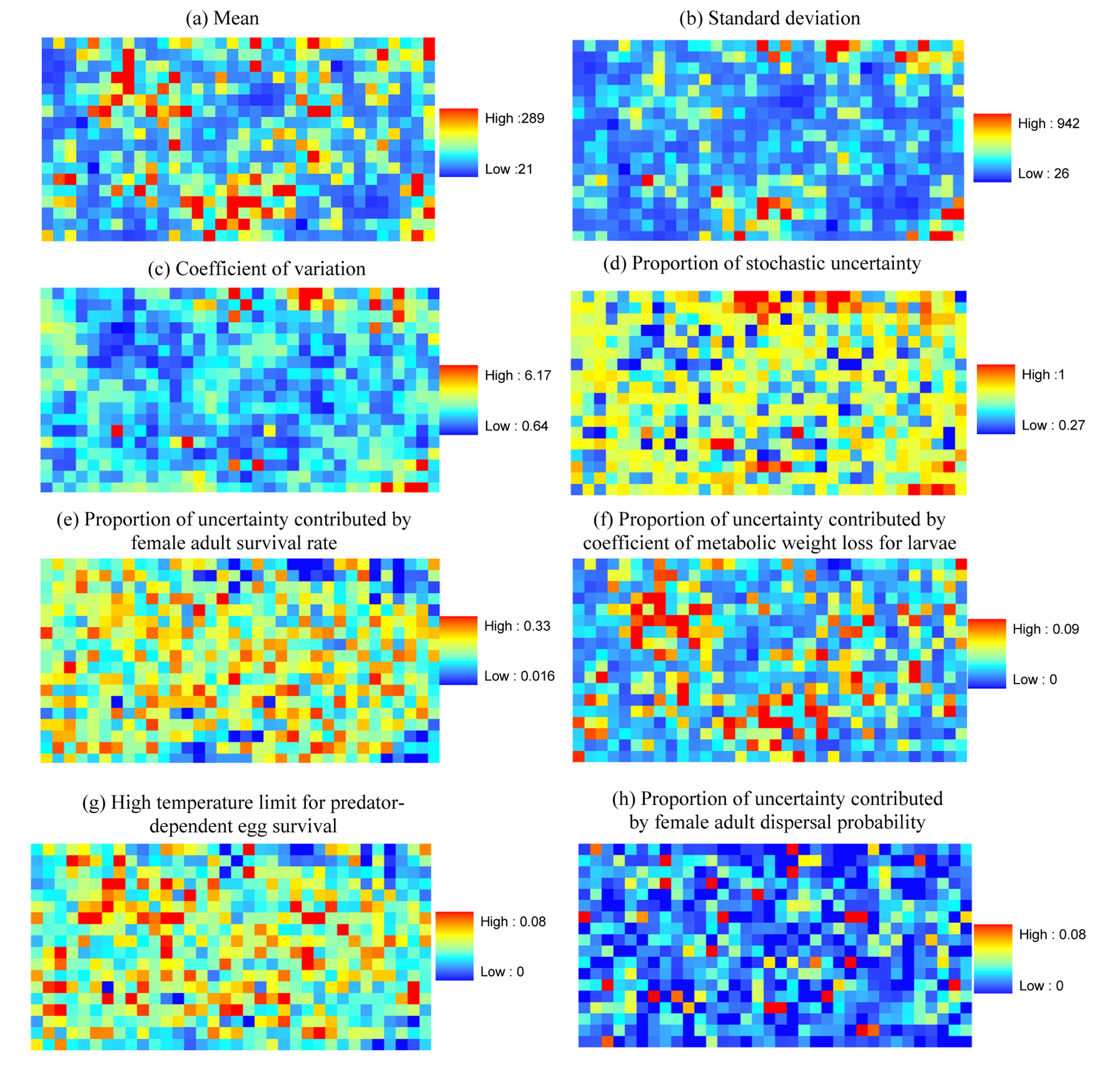

Supplement: Figure S8 — Uncertainty in the predicted larval population density at the individual-house level on simulation day 720. For each individual house, we quantify uncertainty in the population density (as is jointly described by the (a) mean, (b) standard deviation, and (c) coefficient of variation of predicted population density across the parameter sets sampled by FAST), (d) the proportion of uncertainty contributed by stochasticity, and (e–g) the proportions of uncertainty contributed by specific model parameters. To simplify this figure, only parameters with uncertainty contributions in any house larger than 5% are plotted. (1.67 MB TIF) [file pntd.0000830.s015.tif]

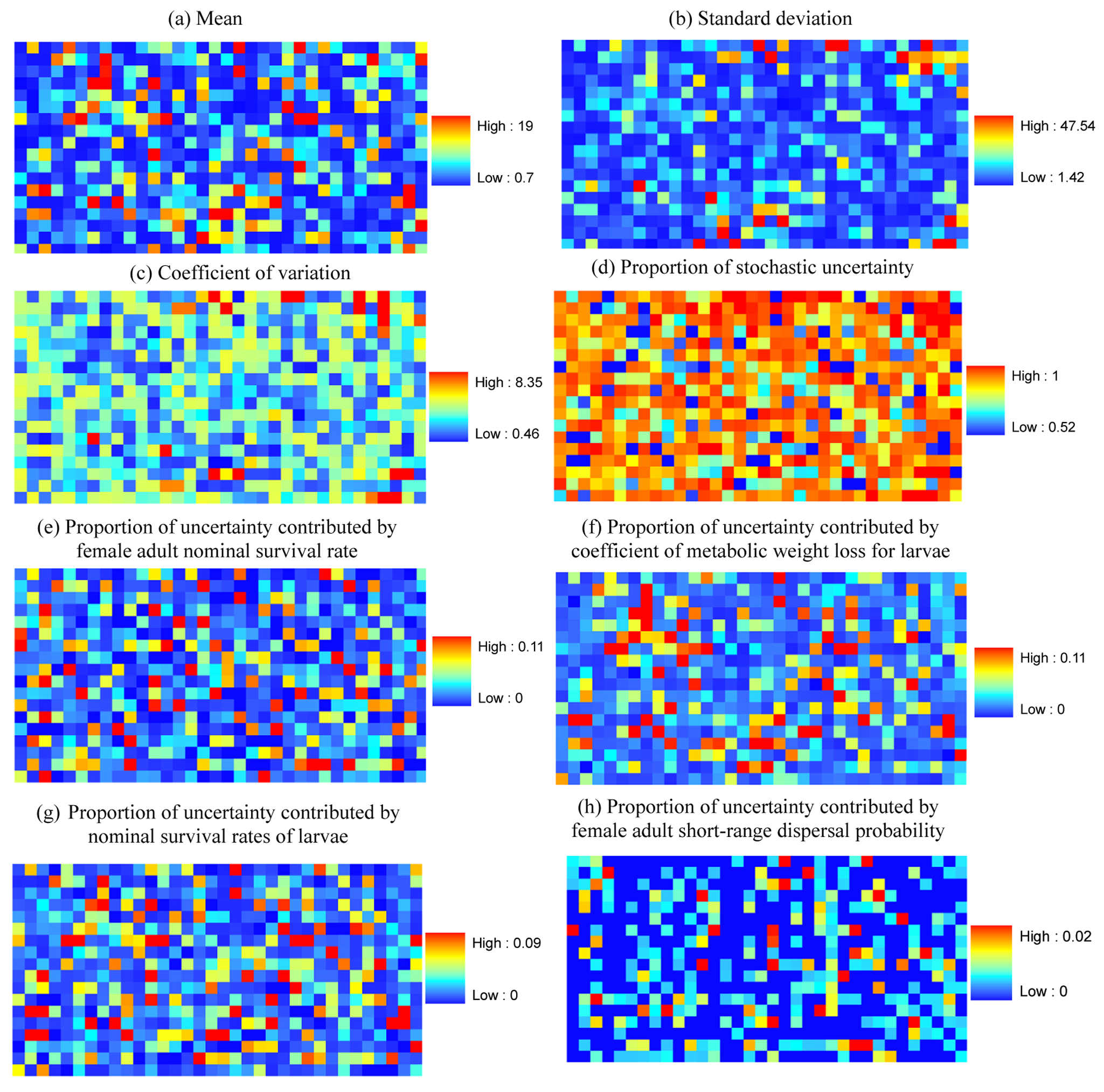

Supplement: Figure S9 — Uncertainty in the predicted pupal density at the individual-house level on simulation day 720. For each individual house, we quantify uncertainty in the predicted population density (as is jointly described by the (a) mean, (b) standard deviation, and (c) coefficient of variation of predicted population density across the parameter sets sampled by FAST), (d) the proportion of uncertainty contributed by stochasticity, and (e–h) the proportions of uncertainty contributed by specific model parameters. To simplify this figure, only parameters with maximum uncertainty contributions larger than 5% in any house are plotted except for panel (h), which is shown for the comparison of mosquito dispersal importance at different life stages. (1.67 MB TIF) [file pntd.0000830.s016.tif]
