# Supplementary material for: Understanding Uncertainties in Model-Based Predictions of Aedes aegypti Population Dynamics
Source: PLoS Negl Trop Dis. 2010 Sep 28;4(9):e830. doi: 10.1371/journal.pntd.0000830 (PMC2946899; doi:10.1371/journal.pntd.0000830)
Supplement: Table S1 — Uncertainties in the estimates of parameters for adults. (0.11 MB DOC) [file pntd.0000830.s017.doc]

Table S1 Uncertainties in the estimates of parameters for adults (15 parameters).

| Parameter | Description | Lower Range | Upper Range | Default  Value | Confidence for  default value | Sources |
| --- | --- | --- | --- | --- | --- | --- |
| *A-FS* | Nominal survival rate for female adults | 0.75 | 0.99 | 0.89 | Moderate | [1,2,3,4,5],, Workshop |
| *A-MS* | Nominal survival rate for male adults | 0.72 | 0.99 | 0.77 | Moderate | [1,2,3,4,5], Workshop |
| *A-TL* | Low temperature limit for nominal  survival (^o^C) | 2 | 10 | 4 | Low | [6], Workshop |
| *A-TH* | High temperature limit for nominal  survival (^o^C) | 35 | 40 | 39 | Low | [6], Workshop |
| *A-TMN* | Minimum temperature for survival (^o^C) | -5 | 2 | 0 | Low | [6,7], Workshop |
| *A-TMX* | Maximum temperature for survival (^o^C) | 40 | 45 | 44 | Low | [6] |
| *A-SDL* | Low saturation deficit limit for  survival (mBar) | 5 | 20 | 10 | Low | [6,7], Workshop |
| *A-SDH* | High saturation deficit limit  for survival (mBar) | 25 | 35 | 30 | Low | [6], Workshop |
| *A-STMN* | Survival factor at the minimum temperature limit for survival | 0 | 0.05 | 0.05 | No | Workshop |
| *A-STMX* | Survival factor at the maximum temperature limit for survival | 0 | 0.05 | 0.05 | No | Workshop |
| *A-SSDH* | Survival factor for saturation deficits higher than *SDH* | 0.55 | 0.95 | 0.6 | Low | [6], Workshop |
| *A-OVTMN* | Minimum temperature for oviposition (^o^C) | 17 | 24 | 18 | Low | [8], Workshop |
| A-F | Coefficient of fecundity (number of eggs per mg wet-weight of female adults) | 35 | 55 | 46.5 | Low | [8] |
| A-DPTG | Development percentage threshold for subsequent gonotrophic cycles | 0.5 | 0.7 | 0.58 | Low | [5,9,10], Workshop |
| A-FWC | Conversion coefficient from dry weight to wet weight for female adults | 1.45 | 1.8 | 1.655 | Low | [8], Workshop |

*Fecundity per gonotrophic cycle is assumed to be linearly related to the wet weight of a female adult. Namely, Fecundity = A-F*×W*_female_, where *W*_female_ is the wet weight of a female adult.

**References:**

1. Maciel-De-Freitas R, Codego CT, Lourenco-De-Oliveira R (2007) Body size-associated survival and dispersal rates of *Aedes aegypti* in Rio de Janeiro. Med Vet Entomol 21: 284-292.

2. McDonald PT (1977) Population characteristics of domestic *Aedes aegypti* (Diptera: Culicidae) in villages on Kenya coast. 1. Adult survival and population size. J Med Entomol 14: 42-48.

3. Sheppard PM, Macdonal.Ww, Tonn RJ, Grab B (1969) Dynamics of an adult population of *Aedes aegypti* in relation to dengue haemorrhagic fever in Bangkok. J Anim Ecol 38: 661-702.

4. Harrington LC, Vermeylen F, Jones JJ, Kitthawee S, Sithiprasasna R, et al. (2008) Age-dependent survival of the dengue vector *Aedes aegypti* (Diptera : Culicidae) demonstrated by simultaneous release-recapture of different age cohorts. J Med Entomol 45: 307-313.

5. Harrington LC, Edman JD, Scott TW (2001) Why do female *Aedes aegypti* (Diptera: Culicidae) feed preferentially and frequently on human blood? J Med Entomol 38: 411-422.

6. Christophers SR (1960) *Aedes aegypti* (L.), the yellow fever mosquito. Cambridge, UK: Cambridge University Press.

7. MacFie JWS (1920) Heat and *Stegomyia fasciata*, short exposures to raised temperatures. Ann Trop Med Parasitol 14: 73-82.

8. Focks DA, Haile DG, Daniels E, Mount GA (1993) Dynamic life table model of *Aedes aegypti* (Diptera: Culicidae) - Analysis of the literature and model development. J Med Entomol 30: 1003-1017.

9. Jalil M (1974) Observations on the fecundity of Aedes triseriatus (Diptera: Culicidae). Entomol Exp Appl 17: 223-233.

10. Nayar JK, Sauerman DM (1975) The effects of nutrition on survival and fecundity in Florida mosquitoes. Part. 3. Utilization of blood and sugar for fecundity. J Med Entomol 12: 220-225.
