# Supplementary material for: Understanding Uncertainties in Model-Based Predictions of Aedes aegypti Population Dynamics
Source: PLoS Negl Trop Dis. 2010 Sep 28;4(9):e830. doi: 10.1371/journal.pntd.0000830 (PMC2946899; doi:10.1371/journal.pntd.0000830)
Supplement: Table S2 — Uncertainties in the estimates of parameters for larvae and pupae. (0.09 MB DOC) [file pntd.0000830.s018.doc]

Table S2 Uncertainties in the estimates of parameters for larvae and pupae (14 parameters).

| Parameter | Description | Lower Range | Upper Range | Default  Value | Confidence for  default value | Sources |
| --- | --- | --- | --- | --- | --- | --- |
| *L-S* | Nominal survival rate for larvae | 0.9 | 1.0 | 0.99 | Low | [1], Workshop |
| *P-S* | Nominal survival rate for pupae | 0.9 | 1.0 | 0.99 | Low | [2], Workshop |
| *LP-TL* | Low temperature limit for nominal survival of larvae and pupae (^o^C) | 10 | 20 | 15 | Moderate | [2], Workshop |
| *LP-TH* | High temperature limit for nominal survival of larvae and pupae (^o^C) | 30 | 40 | 39 | Low | [2,3], Workshop |
| *LP-TMN* | Minimum temperature for survival of larvae and pupae (^o^C) | 5 | 10 | 8 | Moderate | [4], Workshop |
| *LP-TMX* | Maximum temperature for survival of larvae and pupae (^o^C) | 40 | 46 | 44 | Low | Workshop |
| *LP-STMN* | Survival factor at the minimum temperature limit | 0 | 0.05 | 0.05 | No | Workshop |
| *LP-STMX* | Survival factor at the maximum temperature limit | 0 | 0.05 | 0.05 | No | Workshop |
| *P-SEM* | Emergence probability for pupae | 0.75 | 0.9 | 0.83 | Low | [3,4,5], Workshop |
| *LP-SLIP* | Survival of larvae with lipid reserve under fasting | 0.9 | 1 | 0.95 | Low | [6], Workshop |
| *LP-SNLIP* | Survival of larvae without lipid reserve under fasting | 0.3 | 0.7 | 0.5 | Low | [1], Workshop |
| *L-Sp* | Larval survival probability at pupation | 0.9 | 1 | 0.95 | Low | [1], Workshop |
| *L-Wp* | Minimum weight for pupation (mg) | 0.1 | 0.19 | 0.1 | Moderate | [1] |
| *L-SDRY* | Larval survival probability at dry container | 0 | 0.05 | 0.05 | No | Workshop |

**References**

1. Focks DA, Haile DG, Daniels E, Mount GA (1993) Dynamic life table model of *Aedes aegypti* (Diptera: Culicidae) - Analysis of the literature and model development. J Med Entomol 30: 1003-1017.

2. Chang LH, Hsu EL, Teng HJ, Ho CM (2007) Differential survival of *Aedes aegypti* and *Aedes albopictus* (Diptera : Culicidae) larvae exposed to low temperatures in Taiwan. J Med Entomol 44: 205-210.

3. Tsuda Y, Takagi M (2001) Survival and development of *Aedes aegypti* and *Aedes albopictus* (Diptera: Culicidae) larvae under a seasonally changing environment in Nagasaki, Japan. Environ Entomol 30: 855-860.

4. Rueda LM, Patel KJ, Axtell RC, Stinner RE (1990) Temperature-dependent development and survival rates of *Culex quinquefasciatus*  and *Aedes aegypti* (Diptera: Culicidae). J Med Entomol 27: 892-898.

5. Tun-Lin W, Burkot TR, Kay BH (2000) Effects of temperature and larval diet on development rates and survival of the dengue vector *Aedes aegypti* in north Queensland, Australia. Med Vet Entomol 14: 31-37.

6. Southwood TRE, Tonn RJ, Yasuno M, Reader PM, Murdie G (1972) Studies on life budget of *Aedes aegypti* in Wat Samphaya, Bangkok, Thailand. Bull WHO 46: 211-226.
