# Supplementary material for: Understanding Uncertainties in Model-Based Predictions of Aedes aegypti Population Dynamics
Source: PLoS Negl Trop Dis. 2010 Sep 28;4(9):e830. doi: 10.1371/journal.pntd.0000830 (PMC2946899; doi:10.1371/journal.pntd.0000830)
Supplement: Table S3 — Uncertainties in the estimates of parameters for egg survival and hatching. (0.09 MB DOC) [file pntd.0000830.s019.doc]

Table S3 Uncertainties in the estimates of parameters for egg survival and hatching (19 parameters).

| Parameter | Description | Lower Range | Upper  Range | Default  Value | Confidence for  default value | Sources |
| --- | --- | --- | --- | --- | --- | --- |
| *E-S* | Nominal survival rate for eggs | 0.95 | 1.0 | 0.99 | Low | Workshop |
| *E-TL* | Low temperature limit for nominal survival (^o^C) | -6 | 5 | -6 | Low | [1,2], Workshop |
| *E-TH* | High temperature limit for nominal survival (^o^C) | 28 | 35 | 30 | Low | [3], Workshop |
| *E-TMN* | Minimum temperature for survival (^o^C) | -14 | -6 | -14 | Low | [3,4], Workshop |
| *E-TMX* | Maximum temperature for survival (^o^C) | 40 | 45 | 44 | Low | [3] |
| *E-SDL* | Low saturation deficit limit for survival (mBar) | 5 | 20 | 10 | Low | [3,4] |
| *E-SDH* | High saturation deficit limit for survival (mBar) | 25 | 35 | 30 | Low | [3] |
| *E-SE_high_* | High sun exposure limit for survival in dry containers (proportion) | 0.6 | 0.9 | 0.85 | Low | [3], Workshop |
| *E-STMN* | Survival factor at temperatures lower than minimum temperature limit for survival | 0 | 0.05 | 0.05 | No | Workshop |
| *E-STMX* | Survival factor at temperature higher than maximum temperature limit for survival | 0 | 0.05 | 0.05 | No | Workshop |
| *E-SSDH* | Survival factor for saturation deficits higher than *SDH* for containers with low sun exposure | 0.75 | 0.99 | 0.95 | Low | [5], Workshop |
| *E-SSEH* | Survival factor for sun exposure higher than E-*SEH* for dry container | 0.75 | 0.99 | 0.95 | Low | [5], Workshop |
| *E-PTL* | Low temperature limit for predator activities (^o^C) | 15 | 25 | 20 | Low | [5], Workshop |
| *E-PTH* | High temperature limit for predator activities (^o^C) | 25 | 35 | 30 | Low | [3], Workshop |
| *E-SPTL* | Survival factor for predation at low temperatures (< *E-PTL*) | 0.95 | 1 | 0.99 | No | [3,4], Workshop |
| *E-SPTH* | Survival factor for predation at high temperatures (> *E-PTH*) | 0.65 | 0.9 | 0.7 | Low | [5], Workshop |
| *E-HTMN* | Minimum temperature for hatching (^o^C) | 14 | 22 | 22 | Low | [5] Workshop |
| *E-HPNF* | Hatching probability without flooding | 0 | 0.25 | 0.2 | Moderate | [3,6], Workshop |
| *E-HPF* | Hatching probability with flooding | 0.3 | 0.65 | 0.6 | Moderate | [5], Workshop |

**References:**

1. Focks DA, Haile DG, Daniels E, Mount GA (1993) Dynamic life table model for *Aedes aegypti* (Diptera: Culicidae) - Simulation and validation. J Med Entomol 30: 1018-1028.

2. Gilpin ME, McClelland GAH (1979) Systems-analysis of the yellow fever mosquito *Aedes aegypti*. Forts Zool 25: 355-388.

3. Christophers SR (1960) *Aedes aegypti* (L.), the yellow fever mosquito. Cambridge, UK: Cambridge University Press.

4. MacFie JWS (1920) Heat and *Stegomyia fasciata*, short exposures to raised temperatures. Ann Trop Med Parasitol 14: 73-82.

5. Focks DA, Haile DG, Daniels E, Mount GA (1993) Dynamic life table model of *Aedes aegypti* (Diptera: Culicidae) - Analysis of the literature and model development. J Med Entomol 30: 1003-1017.

6. Hien DS (1975) Biology of *Aedes aegypti* (L., 1762) and *Aedes albopictus* (Skuse, 1895) (Diptera, Culicidae) II. Effect Of environmental conditions on the hatching of larvae. Acta Parasitol Pol 23: 537-552
