# Supplementary material for: Understanding Uncertainties in Model-Based Predictions of Aedes aegypti Population Dynamics
Source: PLoS Negl Trop Dis. 2010 Sep 28;4(9):e830. doi: 10.1371/journal.pntd.0000830 (PMC2946899; doi:10.1371/journal.pntd.0000830)
Supplement: Table S4 — Uncertainties in the estimates of parameters for larval weight gain. (0.06 MB DOC) [file pntd.0000830.s020.doc]

Table S4 Uncertainties in the estimates of parameters for larval weight gain (8 parameters).

| Parameter | Description | Lower Range | Upper Range | Default Value | Confidence for default value | Sources |
| --- | --- | --- | --- | --- | --- | --- |
| *Fa* | Conversion rate of consumed  food to biomass | 0.28 | 0.38 | 0.30 | Moderate | Figure 11 in [1], Workshop |
| *Fb* | Exponent of body weight | 0.75 | 0.85 | 0.8 | Moderate | Figure 11 in [1]  Workshop |
| *Fc* | Coefficient of food dependence | 0.05 | 1.0 | 0.1 | No | Figure 11 in [1] |
| *Fd1* | Coefficient of metabolic weight loss | 0.005 | 0.032 | 0.016 | Low | Workshop |
| *Fd2* | Exponent of metabolic activity | 0.59 | 0.73 | 0.667 | Moderate | [1], Workshop |
| *FL_a_* | Intercept for lipid prediction | 0.041 | 0.071 | 0.056 | 0.0073* | Figure 13 in [1] |
| *FL_b_* | Slope for lipid prediction | 0.255 | 0.303 | 0.279 | 0.012 * | Figure 13 in [1] |
| *FF_l_* | Proportion of dead larvae  to liver powder | 0.25 | 0.5 | 0.4 | Low | Figure 15 in [1] |

*Note: For parameters assumed to follow a normal distribution, the “lower range” and “upper range” refer to the 95% confidence interval and the value in the confidence column refers to the estimated standard error.

**References**

1. Gilpin ME, McClelland GAH (1979) Systems-analysis of the yellow fever mosquito *Aedes aegypti*. Forts Zool 25: 355-388.
