# Supplementary material for: Understanding Uncertainties in Model-Based Predictions of Aedes aegypti Population Dynamics
Source: PLoS Negl Trop Dis. 2010 Sep 28;4(9):e830. doi: 10.1371/journal.pntd.0000830 (PMC2946899; doi:10.1371/journal.pntd.0000830)
Supplement: Table S5 — Uncertainties in the estimates of parameters for mosquito dispersal. (0.07 MB DOC) [file pntd.0000830.s021.doc]

Table S5 Uncertainties in the estimates of parameters for mosquito dispersal (7 parameters)

| Parameter | Description | Lower Range | Upper Range | Default Value | Confidence for  default value | Sources |
| --- | --- | --- | --- | --- | --- | --- |
| *SD-FS* | Short-range dispersal probability  for female adults | 0.05 | 0.5 | 0.3 | Low | [1], Workshop |
| *SD-MS* | Short-range dispersal probability  for male adults | 0.05 | 0.5 | 0.3 | Low | [2,3], Workshop |
| *SD-FL* | Long-range dispersal probability  for female adults | 0 | 0.1 | 0.02 | Low | [2,3], Workshop |
| *SD-ML* | Long-range dispersal probability  for male adults | 0 | 0.1 | 0.02 | Low | [3], Workshop |
| *SD-FES* | Short-range dispersal probability for female adults in empty house | 0.1 | 0.9 | 0.8 | Low | [3], Workshop |
| *SD-PES* | Short-range dispersal probability for male adults in empty house | 0.1 | 0.9 | 0.8 | Low | [3], Workshop |
| *SD-LD* | Distance for long range dispersal (house-distance) | 5 | 20 | 10 | Low | [3], Workshop |

**References:**

1. Gilpin ME, McClelland GAH (1979) Systems-analysis of the yellow fever mosquito *Aedes aegypti*. Forts Zool 25: 355-388.

2. Harrington LC, Scott TW, Lerdthusnee K, Coleman RC, Costero A, et al. (2005) Dispersal of the dengue vector *Aedes aegypti* within and between rural communities. Am J Trop Med Hyg 72: 209-220.

3. Magori K, Legros M, Puente ME, Focks DA, Scott TW, et al. (2009) Skeeter Buster: a stochastic, spatially-explicit modeling tool for studying *Aedes aegypti* population replacement and population suppression strategies. Plos Neglect Trop Dis 3: e508.
