# Supplementary material for: Understanding Uncertainties in Model-Based Predictions of Aedes aegypti Population Dynamics
Source: PLoS Negl Trop Dis. 2010 Sep 28;4(9):e830. doi: 10.1371/journal.pntd.0000830 (PMC2946899; doi:10.1371/journal.pntd.0000830)
Supplement: Table S6 — Uncertainty contributions (%) by different model parameters for predicted egg population density at the community level. (0.05 MB DOC) [file pntd.0000830.s022.doc]

Table S6 Uncertainty contributions (%) by different model parameters for predicted egg

population density at the community level

| Parameters | Descriptions | Uncertainty contribution | Standard error |
| --- | --- | --- | --- |
| *A-FS* | Nominal daily survival rate for female adults | 71.75 | 2.42 |
| *A-MS* | Nominal daily survival rate for male adults | 6.56 | 0.55 |
| *A-F* | Coefficient of fecundity for female adults | 2.60 | 0.34 |
| *L-S* | Nominal daily survival rate for larvae | 2.56 | 0.34 |
| *Fc* | Coefficient of food dependence for larvae | 1.75 | 0.28 |
| *A-D* | Gonotrophic development rate | 1.65 | 0.27 |
| *P-S* | Nominal daily survival rate for pupae | 1.31 | 0.24 |
| *A-FWC* | Conversion coefficient from dry weight to wet weight for female adults | 1.06 | 0.21 |

Note: Only parameters that contribute more than one percent to the uncertainty are shown in the table. They explain 89.2% of uncertainty in the predicted population density.
