# Supplementary material for: Understanding Uncertainties in Model-Based Predictions of Aedes aegypti Population Dynamics
Source: PLoS Negl Trop Dis. 2010 Sep 28;4(9):e830. doi: 10.1371/journal.pntd.0000830 (PMC2946899; doi:10.1371/journal.pntd.0000830)
Supplement: Table S7 — Uncertainty contributions (%) by different model parameters for predicted larval population density at the community level. (0.05 MB DOC) [file pntd.0000830.s023.doc]

Table S7 Uncertainty contributions (%) by different model parameters for predicted

larval population density at the community level

| Parameters | Descriptions | Uncertainty contribution | Standard error |
| --- | --- | --- | --- |
| *A-FS* | Nominal daily survival rate for female adults | 39.42 | 1.54 |
| *E-PTH* | High temperature limit for predator activities on eggs | 9.48 | 0.67 |
| *Fd1* | Coefficient of metabolic weight loss for larvae | 7.66 | 0.59 |
| *L-S* | Nominal daily survival rate for larvae | 5.60 | 0.50 |
| *A-MS* | Nominal daily survival rate for male adults | 4.27 | 0.43 |
| *E-SPTH* | Survival factor of predation at high temperatures for egg | 3.73 | 0.40 |
| *E-TH* | High temperature limit for nominal egg survival | 3.09 | 0.37 |
| *E-PTL* | Low temperature limit for predator activities on eggs | 2.79 | 0.35 |
| *E-D* | Embryonic development rate | 1.79 | 0.28 |
| *P-S* | Nominal daily survival rate for pupae | 1.38 | 0.24 |
| *A-D* | Gonotrophic development rate | 1.05 | 0.21 |

Note: Only parameters that contribute more than one percent to the uncertainty are shown in the table. They explain 80.3% of uncertainty in the predicted population density.
