# Supplementary material for: Understanding Uncertainties in Model-Based Predictions of Aedes aegypti Population Dynamics
Source: PLoS Negl Trop Dis. 2010 Sep 28;4(9):e830. doi: 10.1371/journal.pntd.0000830 (PMC2946899; doi:10.1371/journal.pntd.0000830)
Supplement: Table S8 — Uncertainty contributions (%) by different model parameters for predicted pupal population density at the community level. (0.05 MB DOC) [file pntd.0000830.s024.doc]

Table S8 Uncertainty contributions (%) by different model parameters for predicted

pupal population density at the community level

| Parameters | Descriptions | Uncertainty contribution | Standard error |
| --- | --- | --- | --- |
| *Fd1* | Coefficient of metabolic weight loss for larvae | 20.50 | 1.05 |
| *L-S* | Nominal daily survival rate for larvae | 17.37 | 0.95 |
| *A-FS* | Nominal daily survival rate for female adults | 14.18 | 0.85 |
| *L-D* | Larval development rate | 7.14 | 0.58 |
| *E-PTH* | High temperature limit for predator activities on eggs | 3.72 | 0.41 |
| *P-D* | Pupal development rate | 3.23 | 0.38 |
| *P-S* | Nominal daily survival rate for pupae | 3.23 | 0.38 |
| *Fa* | Conversion rate of consumed food to biomass for larvae | 2.91 | 0.36 |
| *A-MS* | Nominal daily survival rate for male adults | 1.61 | 0.25 |
| *Fc* | Coefficient of food dependence for larvae | 1.34 | 0.24 |
| *E-SPTH* | Survival factor for predation at high temperatures | 1.21 | 0.23 |

Note: Only parameters that contribute more than one percent to the uncertainty are shown in the table. They explains 77.6% of uncertainty in the predicted population density.
