# Supplementary material for: Understanding Uncertainties in Model-Based Predictions of Aedes aegypti Population Dynamics
Source: PLoS Negl Trop Dis. 2010 Sep 28;4(9):e830. doi: 10.1371/journal.pntd.0000830 (PMC2946899; doi:10.1371/journal.pntd.0000830)
Supplement: Table S9 — Uncertainty contributions (%) by different model parameters for the predicted population density of nulliparous female adults at the community level. (0.05 MB DOC) [file pntd.0000830.s025.doc]

Table S9 Uncertainty contributions (%) by different model parameters for the predicted

population density of nulliparous female adults at the community level

| Parameters | Descriptions | Uncertainty contribution | | Standard error |  |
| --- | --- | --- | --- | --- | --- |
| *A-FS* | Nominal daily survival rate for female adults | | 24.50 | 1.17 |  |
| *L-S* | Nominal daily survival rate for larvae | | 17.84 | 0.97 |  |
| *Fd1* | Coefficient of metabolic weight loss for larvae | | 16.42 | 0.92 |  |
| *L-D* | Larval development rate | | 6.06 | 0.53 |  |
| *P-S* | Nominal daily survival rate for pupae | | 5.51 | 0.50 |  |
| *E-PTH* | High temperature limit for predator activities on eggs | | 2.52 | 0.33 |  |
| *A-MS* | Nominal daily survival rate for male adults | | 2.39 | 0.31 |  |
| *Fa* | Conversion rate of consumed food to biomass for larvae | | 2.28 | 0.32 | |
| *P-SEM* | Emergence probability for pupae | | 2.28 | 0.32 | |
| *A-D* | Gonotrophic development rate | | 1.10 | 0.22 | |
| *L-Sp* | Larval survival probability at pupation | | 1.09 | 0.22 | |

Note: Only parameters that contribute more than one percent to the uncertainty are shown in the table. They explains 82% of uncertainty in the predicted population density.
