# Supplementary material for: Understanding Uncertainties in Model-Based Predictions of Aedes aegypti Population Dynamics
Source: PLoS Negl Trop Dis. 2010 Sep 28;4(9):e830. doi: 10.1371/journal.pntd.0000830 (PMC2946899; doi:10.1371/journal.pntd.0000830)
Supplement: Table S10 — Uncertainty contributions (%) by different model parameters for the predicted population density of parous female adults at the community level. (0.04 MB DOC) [file pntd.0000830.s026.doc]

Table S10 Uncertainty contributions (%) by different model parameters for the predicted

population density of parous female adults at the community level

| Parameters | Descriptions | Uncertainty contribution | Standard error |
| --- | --- | --- | --- |
| *A-FS* | Nominal daily survival rate for female adults | 69.82 | 3.88 |
| *A-MS* | Nominal daily survival rate for male adults | 6.16 | 0.60 |
| *L-S* | Nominal daily survival rate for larvae | 3.51 | 0.42 |
| *Fd1* | Coefficient of metabolic weight loss for larvae | 2.85 | 0.38 |
| *L-D* | Larval development rate | 1.48 | 0.26 |
| *P-S* | Nominal daily survival rate for pupae | 1.00 | 0.21 |

Note: Only parameters that contribute more than one percent to the uncertainty are shown in the table. They explains 84.8% of uncertainty in the predicted population density.
