# Supplementary material for: Understanding Uncertainties in Model-Based Predictions of Aedes aegypti Population Dynamics
Source: PLoS Negl Trop Dis. 2010 Sep 28;4(9):e830. doi: 10.1371/journal.pntd.0000830 (PMC2946899; doi:10.1371/journal.pntd.0000830)
Supplement: Table S11 — Uncertainty contributions (%) by different model parameters for the predicted population density of male adults at the community level. (0.05 MB DOC) [file pntd.0000830.s027.doc]

Table S11 Uncertainty contributions (%) by different model parameters for the predicted

population density of male adults at the community level

| Parameters | Descriptions | Uncertainty contribution | Standard error |
| --- | --- | --- | --- |
| *A-MS* | Nominal daily survival rate for male adults | 29.09 | 1.38 |
| *A-FS* | Nominal daily survival rate for female adults | 18.32 | 1.02 |
| *Fd1* | Coefficient of metabolic weight loss for larvae | 13.51 | 0.85 |
| *L-S* | Nominal daily survival rate for larvae | 10.82 | 0.74 |
| *L-D* | Larval development rate | 4.29 | 0.44 |
| *P-S* | Nominal daily survival rate for pupae | 4.27 | 0.44 |
| *E-PTH* | High temperature limit for predator activities on eggs | 3.11 | 0.36 |
| *P-SEM* | Emergence probability for pupae | 2.14 | 0.31 |
| *Fa* | Conversion rate of consumed food to biomass for larvae | 1.79 | 0.28 |
| *Fc* | Coefficient of food dependence for larvae | 1.06 | 0.21 |

Note: Only parameters that contribute more than one percent to the uncertainty are shown in the table. They explains 88.4% of uncertainty in the predicted population density.
